# High‐Resolution 3D Printing for Electronics

**DOI:** 10.1002/advs.202104623

**Published:** 2022-01-17

**Authors:** Young‐Geun Park, Insik Yun, Won Gi Chung, Wonjung Park, Dong Ha Lee, Jang‐Ung Park

**Affiliations:** ^1^ Department of Materials Science and Engineering Yonsei University Seoul 03722 Republic of Korea; ^2^ Center for Nanomedicine Institute for Basic Science (IBS) Seoul 03722 Republic of Korea; ^3^ Graduate Program of Nano Biomedical Engineering (NanoBME) Advanced Science Institute Yonsei University Seoul 03722 Republic of Korea

**Keywords:** 3D printings, freeform electronics, integrated circuits, printed electronics, wireless sensors

## Abstract

The ability to form arbitrary 3D structures provides the next level of complexity and a greater degree of freedom in the design of electronic devices. Since recent progress in electronics has expanded their applicability in various fields in which structural conformability and dynamic configuration are required, high‐resolution 3D printing technologies can offer significant potential for freeform electronics. Here, the recent progress in novel 3D printing methods for freeform electronics is reviewed, with providing a comprehensive study on 3D‐printable functional materials and processes for various device components. The latest advances in 3D‐printed electronics are also reviewed to explain representative device components, including interconnects, batteries, antennas, and sensors. Furthermore, the key challenges and prospects for next‐generation printed electronics are considered, and the future directions are explored based on research that has emerged recently.

## Graphical abstract

Recent remarkable advances in 3D‐printable functional materials and high‐resolution 3D printing methods to fabricate freeform electronics are discussed in this review as their latest applications in various device components, with perspectives on the future direction of 3D printing technologies.

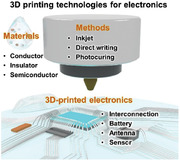

## 1 Introduction

Form factor, in terms of engineering and design, refers to the structured physical specification of product such as size and shape.^[^ ^1^ ^]^ Diversification in the structure of electronic devices has led to increasing demands for the free form‐factor (i.e., freeform) architecture in emerging electronics including wearable electronics, bioelectronics, optoelectronics, batteries, and soft robotics.^[^ ^2^, ^3^, ^4^, ^5^, ^6^, ^7^ ^]^ Also, the demands for high‐performance, miniaturized electronic devices have led to the exponential growth in the density of electronic chips and the complexity of their integration.^[^ ^8^, ^9^ ^]^ Therefore, electronics have been required to have 3D form with occupying *z*‐axis space while maintaining the miniaturized areal dimension.^[^ ^10^ ^]^ To satisfy these demands in the current fabrication process, additive manufacturing, such as 3D printing, has been introduced and developed for fabricating freeform 3D structures with high resolution.

The development of strategies for forming 3D structures in certain electronics is of increasing interest, including the potential for creating new characteristics and enhanced functionalities.^[^ ^11^ ^]^ 3D microstructures and nanostructures of functional materials can modify the sensitivity of sensors and the capacity of energy storage devices drastically by increasing their effective surface areas.^[^ ^12^, ^13^, ^14^ ^]^ In addition, recent applications of electronics in biomedical engineering and the Internet‐of‐Things (IoT) expand the form factors of devices with conformal 3D designs to non‐planar biological surfaces and objects.^[^ ^15^ ^]^ For example, current research in the field of wearable electronics essentially is focused on forming the structures of these electronic devices to be conformally interfaced with target surfaces such as skins and eyes,^[^ ^16^, ^17^, ^18^ ^]^ to be efficiently mounted and to transfer the signals with living organisms.

The use of photolithography‐based processes is the conventional method of modeling and fabricating electronics. Although this method, which was developed in the previous century, significantly contributed to increasing the integrity of electronics with scalability, it only can create 2D structures on flat surfaces, which makes it difficult to fully implement them in cutting‐edge electronics that have complicated 3D geometries. Numerous 3D printing methods, such as material jetting, extrusion, polymerization, fusion, and sintering, have been researched to form 3D structures of various material groups, including insulators, semiconductors, and conductors, to provide 3D‐printed electronics. As a result, remarkable research progress has been achieved in the integration, complexity, performance, and applicability of electronic devices.

**Figure 1:**
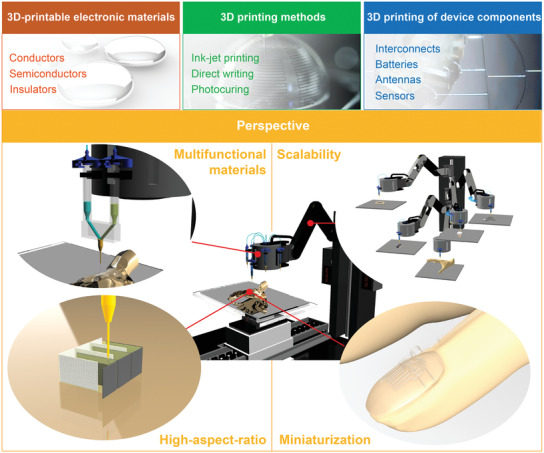
Summary of the 3D printing methods, materials and applications for freeform electronics with multifunctional materials, high aspect ratio structures, miniaturization, and scalability.

In this review, as illustrated in **Figure** **1**, we discuss the recent advances in the research associated with high‐resolution 3D printing technologies for the formation of freeform electronics, and our focus is on printable electronic materials and their 3D printing methods. The representative applications of 3D printing in electronics, including interconnects, energy storage devices, radiofrequency (RF) devices, and sensors, are discussed in terms of their ink materials and printing methods. We also review the additional requirements in 3D printing technologies for achieving high‐performance, integrated electronics. These requirements include promising frontiers, such as scalability, the ability to form miniaturized and high‐aspect‐ratio structures, and the ability to print multiple materials. The challenges, opportunities, and perspectives for further innovation of 3D‐printed electronics also are discussed.

## 2 3D‐Printable Electronic Materials

### 2.1 Conductors

Photographic patterning methods have contributed to the formation of a variety of 2D conductive traces in conventional electronics. For the further miniaturization and complexification of the electronic device and its components, it will be necessary to develop freeform 3D conductive structures with high resolutions and minimized displacement errors for their integration. Therefore, numerous materials with electrically conductive properties have been developed as ink for 3D printing.^[^ ^19^ ^]^ Metallic materials are representative conductors for electronic devices due to their high electrical conductivity. However, bulk metal is a hard solid that has a high melting point, and this prohibits the formation of the metal as a liquid‐phase ink.

**Figure 2:**
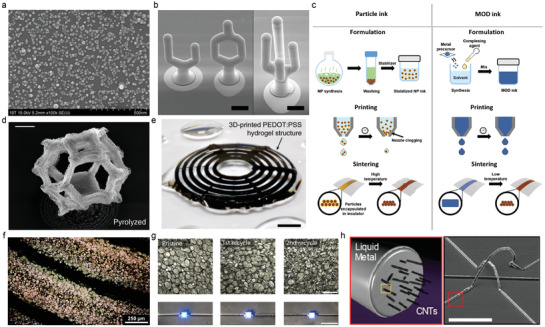
Conductors as forms of printable ink. a) SEM of the synthesized Ag nanoparticles. b) SEM of the high‐aspect‐ratio structures fabricated with inkjet‐printed gold nanoparticles. Scale bar, 100 µm. c) Schematic illustration of preparation processes and working mechanisms of the metallic particle ink and MOD ink. d) SEM image of the tetrakaidecahedron unit cell after pyrolysis. Scale bar, 4 µm. e) Photograph of a 3D‐printed conducting polymer structure with overhanging features in a hydrogel state. Scale bar, 2 mm. f) Optical micrograph of the printed liquid metal‐elastomer. g) Optical micrographs of the liquid metal droplet microstructure with recycling (top), and photographs of the LED with recycling (bottom). Scale bars, 100 µm (top), and 10 mm (bottom). h) Left: The schematic illustration of the CNT/LM composite, Right: SEM image of the 3D structure of the CNT/LM composite. Scale bar, 100 µm. a) Reproduced with permission.^[^ ^24^ ^]^ Copyright 2012, American Chemical Society. b) Reproduced with permission.^[^ ^26^ ^]^ Copyright 2014, Wiley‐VCH. c) Reproduced with permission.^[^ ^36^ ^]^ Copyright 2018, Royal Society of Chemistry. d) Reproduced with permission.^[^ ^37^ ^]^ Copyright 2018, Springer Nature. e) Reproduced with permission.^[^ ^44^ ^]^ Copyright 2020, Springer Nature. f) Reproduced with permission.^[^ ^54^ ^]^ Copyright 2020, Royal Society of Chemistry. g) Reproduced with permission.^[^ ^55^ ^]^ Copyright 2021, Springer Nature. h) Reproduced with permission.^[^ ^56^ ^]^ Copyright 2019, American Chemical Society.

The metallic nanoparticle ink, a suspension with metal nanoparticles and liquid medium, exhibits a high surface‐to‐volume ratio, and this feature resulted in a decrease of post‐treatment temperature (**Figure** **2**a).^[^ ^20^, ^21^, ^22^, ^23^, ^24^ ^]^ Polymer encapsulation with organic additives and stabilizing agents is used to prevent the agglomeration of these nanoscale particles inside the liquid medium by the Van der Waals interaction. As the electron conduction can be disturbed by the surrounding polymers, post‐treatment, such as the sintering process, is required to remove the polymer medium and to recover the conductivity between the particles.

Metallic nanoparticle inks are advantageous for the diversity of applicable ink materials. The change in the metal and the liquid medium can apply various characteristics to the ink. To achieve high stretchability, Huang et al. developed an Ag nanoparticle ink by blending Ag nanoparticles with polyacrylic acid (PAA), carboxymethylcellulose sodium (CMC), hybrid solvents, and sodium chloride (NaCl) solution.^[^ ^25^ ^]^ The chlorides triggered the room‐temperature sintering of the Ag nanoparticles and enabled the viscous ink to exhibit a high conductivity after drying at only 80 °C. The addition of CMC promotes the formation of hydrogen bonds with PAA, which ensures that the sintered Ag aggregates keep in close contact with each other under mechanical strain during stretching.

The resolution of metallic nanoparticle‐based ink can be improved by modifying the sintering strategies. Sadie et al. directly dropped Au nanoparticle inks from a nozzle to a high‐temperature substrate to print and sinter the ink simultaneously.^[^ ^26^ ^]^ A pillar‐shaped 3D nanostructure with an aspect ratio of 6:1 was formed by about 50 droplets. Also, complex structures such as vertical polygons were 3D‐printed with high resolution (Figure 2b). When sintered at 200 °C, electrical and mechanical performances were achieved that were superior to the performances of conventional lead‐tin eutectic materials. The laser‐assisted sintering method can produce high‐resolution 3D structures by selective sintering at the central part of the 3D‐printed nanoparticles.^[^ ^27^, ^28^ ^]^ The laser‐assisted sintering also decreased the oxidation of metallic nanoparticles by reducing the sintering time.

The high‐temperature sintering process of the metallic nanoparticle ink limits the printing to a substrate that is vulnerable to heat (e.g., plastic, polymer film). To avoid these complications, various printing techniques and inks with various components to lower the sintering temperature, such as photocuring polymers, have been developed.^[^ ^29^ ^]^ Tobjörk et al. developed an Ag nanoparticle ink with 20 wt% concentration dispersed into the ethanol and ethylene glycol solvents.^[^ ^30^ ^]^ Ink‐jet printing was used to print the ink on a paper substrate. After thermal annealing at low temperature, that is, <120 °C, additional infrared (IR) lamp sintering for 15 s was done to form a printed ink with a volume resistance of 10 µΩ cm, which is about 10–20% of the resistance of bulk silver. Saleh et al. developed a 3D‐printable ink with Ag nanoparticles and sintered the ink with ultraviolet (UV) light.^[^ ^31^ ^]^

The aggregates formed by the agglomeration between metallic nanoparticles can limit high‐resolution printing by clogging the microfluidic system of a printer.^[^ ^32^ ^]^ To overcome these disadvantages, particle‐free metallic‐organic decomposition (MOD) inks were developed in which metal precursors were mixed with complexing agents. MOD inks usually contain lower concentrations of metal than metallic nanoparticle inks, but the omission of nanoparticle processing makes it cost‐effective.^[^ ^33^, ^34^, ^35^ ^]^ Compared with the metallic nanoparticle inks, sintering at low temperatures was achieved because there were no colloidal stabilizing agents. This prevents the oxidation of metallic particles, which occurs when the high‐temperature sintering is done.^[^ ^36^ ^]^ Figure 2c compares metallic nanoparticle ink and MOD ink. Vyatskikh et al. synthesized a metal‐rich photoresist with nickel acrylate by a ligand exchange reaction between nickel alkoxide and acrylic acid, combined with an acrylic monomer (i.e., pentaerythritol triacrylate) and a photo‐initiator (i.e., 7‐diethylamino‐3‐thenoylcoumarin).^[^ ^37^ ^]^ This was drop cast to a silicon substrate, and the two‐photon lithography method was used to fabricate a freeform 3D nanoarchitecture. The organic compounds were removed by pyrolysis to form a Ni architecture with a high concentration of 90 wt% and a small unit cell width of a few micrometers (Figure 2d).

Currently, the development of wearable bioelectronic devices has created interest in free‐formable inks that are electromechanically stable and biocompatible. 3D‐printable inks have been developed for soft, stretchable conductors, using carbon‐based materials (e.g., carbon nanotube (CNT),^[^ ^38^, ^39^, ^40^ ^]^ and graphene^[^ ^41^, ^42^ ^]^) and polymer‐based materials (e.g., poly(3,4‐ethylene dioxythiophene):polystyrene sulfonate (PEDOT:PSS)). These inks are conductive, stable, and stretchable. Mixing the carbon‐based materials into various media (polymers, liquid metals, and others) enables the maintenance of the characteristics of the medium, and it does not interfere with the electrical conductance of the carbon‐based materials. Liao et al. developed a carbon‐based ink by using a multi‐walled carbon nanotube/carbon black/graphite composite as a conductive filler and using acrylic resin as a binder.^[^ ^43^ ^]^ This highly conductive ink had a sheet resistance of 29 Ω sq^−1^ with a line thickness of 40 µm. Yuk et al. developed a PEDOT:PSS hydrogel ink suitable for direct writing.^[^ ^44^ ^]^ The aqueous PEDOT:PSS was frozen cryogenically and lyophilized, and it was re‐dispersed into water that contained 5–7 wt% dimethyl sulfoxide (DMSO) to form a paste‐like, conducting polymer ink. The PEDOT:PSS ink exhibited the minimum line width of about 30 µm with electrical conductivities as high as 155 S cm^−1^ in the dry state and 28 S cm^−1^ in the hydrogel state, comparable to the previously reported high‐performance conducting polymers (Figure 2e).

Gallium‐based liquid metals, such as eutectic gallium‐indium alloy (EGaIn), exist in the liquid phase at room temperature, and they have high electrical conductivity.^[^ ^45^, ^46^, ^47^ ^]^ They are easily deformable, self‐healable, and dispersible, and these characteristics make it possible to use them in various fields, such as stretchable circuits, strain sensors, and wearable electronics.^[^ ^48^, ^49^, ^50^, ^51^ ^]^ Also, liquid metals can maintain their 3D structure by the formation of a solid‐phase oxide layer at the surface, which makes the liquid metal suitable for printable ink in 3D printing.^[^ ^52^, ^53^ ^]^ Neumann et al. fabricated a particle made of representative gallium‐based liquid metals, and then formed a suspension with 90 wt% concentration by mixing EGaIn into a poly(dimethyl siloxane) (PDMS) medium.^[^ ^54^ ^]^ This suspension ink was 3D‐printed to the substrate using direct writing (Figure 2f). The dispersed EGaIn nanodroplets inside the PDMS medium agglomerated and created a path that conducted electricity when pressed by local pressure, with no need for a heating process. Tutika et al. developed recyclable characteristics and produced conventional self‐healing, reconfigurable liquid metal ink by mixing the styrene‐isoprene‐styrene block copolymer and the polybutadiene plasticizer to form an elastomer‐plasticizer‐liquid metal composite.^[^ ^55^ ^]^ When a compressive load was applied to the composite, the liquid metal droplets, which were insulated by the polymer, agglomerated to form an electrically conductive network. It was confirmed that the electrical conductivity and the microstructure can be maintained even after two recycling processes (Figure 2g). There also is a method to improve properties by mixing additives with a liquid metal to form a composite. Park et al. increased the mechanical strength and structural stability of liquid metal by forming a composite of CNT and EGaIn liquid metal (CNT/LM) (Figure 2h).^[^ ^56^ ^]^ The enhanced structural stability of the liquid metal was suitable for the freeform interfaces of electronics.

### 2.2 Semiconductors

**Figure 3:**
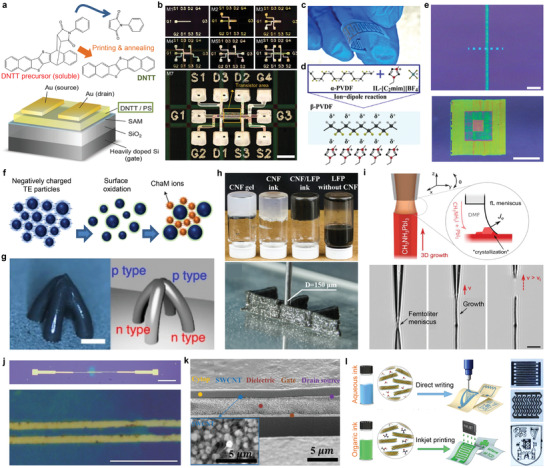
Semiconductors as forms of printable ink. a) Top: chemical structure of the soluble DNTT precursor and its thermal conversion process. Bottom: Schematic illustration of the device structure of DNTT TFT. b) Sequential images of the process of printing seven metal layers. Scale bar, 1 mm. c) A photograph of the printed PVDF‐TrFE piezoelectric film. d) Schematic illustration of PVDF inks. e) Optical micrographs 3D‐printed single line pattern of organic semiconductors, and the multilayer printing. Scale bars, 10 µm and 100 µm, respectively. f) Schematic illustration showing the design of thermoelectric ink. g) Photograph and illustrated premodels of arch‐type architectures consisting of junctional p‐ and n‐type inorganic semiconductors. Scale bar, 500 µm. h) Top: photograph of CNF‐containing inks and pure LiFePO_4_ (LFP) dispersion ink, Bottom: photograph of 3D‐printed CNF/LFP structure. i) Top: schematic illustration showing 3D printing of halide perovskites. Bottom: optical micrographs showing the 3D printing processes. Scale bar, 10 µm. j) Optical micrographs of rGO TFTs. Scale bars, 200 µm and 30 µm, respectively. k) Cross‐sectional SEM of printed CNT TFT. Inset: magnified SEM of printed CNT layer. l) Schematic illustration and photographs of printed Mxene ink. a) Reproduced with permission.^[^ ^58^ ^]^ Copyright 2015, Wiley‐VCH. b) Reproduced with permission.^[^ ^60^ ^]^ Copyright 2019, Springer Nature. c) Reproduced with permission.^[^ ^61^ ^]^ Copyright 2021, Elsevier. d) Reproduced with permission.^[^ ^62^ ^]^ Copyright 2021, American Chemical Society. e) Reproduced under the terms of the Creative Commons CC‐BY license.^[^ ^6^ ^]^ Copyright 2019, The Authors. Published by Wiley‐VCH. f,g) Reproduced with permission.^[^ ^65^ ^]^ Copyright 2021, Springer Nature. h) Reproduced with permission.^[^ ^66^ ^]^ Copyright 2019, Wiley‐VCH. i) Reproduced with permission.^[^ ^67^ ^]^ Copyright 2019, Wiley‐VCH. j) Reproduced with permission.^[^ ^73^ ^]^ Copyright 2015, Wiley‐VCH. k) Reproduced with permission.^[^ ^74^ ^]^ Copyright 2020, Wiley‐VCH. l) Reproduced with permission.^[^ ^75^ ^]^ Copyright 2019, Springer Nature.

Semiconductors modulate their electrical properties in response to external conditions such as mechanical stresses, chemical adsorptions, and electric fields. Conversely, they can also change their physical properties through electrical control. Due to these functionalities, semiconductors are essential for various electronic devices including transistors and sensors. Although silicon or oxide‐based inorganic semiconductor materials have been used in conventional high‐performance electronics by the photolithography‐based subtractive fabrication process, organic semiconducting materials were actively researched as 3D‐printable inks due to their facile deformability and solubility to various organic solvents.^[^ ^57^ ^]^ Kimura et al. demonstrated the printable ink using a soluble precursor of dinaphtho[2,3‐b:2′,3′‐f]thieno[3,2‐b]thiophene (DNTT) in chloroform (**Figure** **3**a).^[^ ^58^ ^]^ To enhance the wettability of ink to the substrates, polystyrene was blended with the precursor. The printed inks were annealed at 200 °C, and the printed thin‐film transistor (TFT) with the DNTT channel exhibited field‐effect mobilities of 1.1 cm^2^ V^−1^ s^−1^. Niazi et al. introduced the printing of organic TFTs using 2,8‐difluoro‐5, 11‐bis(triethylsilylethynyl) anthradithiophene (diF‐TES‐ADT) as an organic semiconductor, with blending polystyrene or poly(alpha‐methyl styrene) for improving the printing quality.^[^ ^59^ ^]^ The fabricated TFT showed field‐effect mobilities of 6.7 cm^2^ V^−1^ s^−1^. Kwon et al. demonstrated the direct printing of multilayer TFTs (Figure 3b).^[^ ^60^ ^]^ Dual‐gate structure for complementary organic semiconductors was printed with benzobis(thiadiazole) derivative (TU‐3) and dithieno[2,3‐d;2′,3′‐d′]benzo[1,2‐b;4,5‐b′]dithiophene (DTBDT‐C6), as n‐type and p‐type organic semiconductors, respectively. Yuan et al. printed poly(vinylidene fluoride‐*co*‐trifluoro ethylene) PVDF‐TrFE, which is a representative polymeric piezoelectric material.^[^ ^61^ ^]^ A mixture of dimethyl sulfoxide and acetone was used as a solvent system for PVDF‐TrFE, and a piezoelectric nanogenerator was demonstrated (Figure 3c).

As organic materials have a relatively low melting point, some approaches used elevated temperatures to melt the materials without solvent. Liu et al. introduced the 3D printing of PVDF by melting it by 200 °C heaters at the front of the nozzle.^[^ ^62^ ^]^ By mixing the ionic liquid 1‐ethyl‐3‐methylimidazolium tetrafluoroborate ((C2mim)(BF_4_)) with PVDF, the printed PVDF showed high crystallinity with piezoelectric *β*‐phase (Figure 3d).

The high solubility and low melting points of organic semiconducting materials can take advantage in miniaturizing the scale of printed patterns. An et al. demonstrated 3D printings of semiconducting materials for organic light‐emitting diodes (OLEDs).^[^ ^6^ ^]^ Three kinds of small‐molecule or polymeric organic materials were dissolved into organic solvents such as water or chlorobenzene, and printed using a nozzle with an inner diameter of 2 µm. The printed line widths of each material were below 5 µm, and small droplet volume facilitated multilayer printing due to rapid evaporation of solvents (Figure 3e).

Generally, inorganic semiconductors have higher performance and stability compared to organic semiconductors. Accordingly, research on producing inorganic semiconductor materials in the form of printable ink was conducted. Kim et al. presented the high‐resolution printing of indium oxide semiconductor, using the precursor solution of indium nitrate hydrate (In(NO_3_)_3_‐*x*H_2_O) in deionized water.^[^ ^63^ ^]^ Due to the precursor‐type inks with no solid contents, The indium oxide channels for TFT were printed with 2 µm of line width. The printed TFTs showed mobility of 230 cm^2^ V^−1^ s^−1^ after annealing at 250 °C. Similarly, Scheideler et al. demonstrated the printing of indium oxide transistors with a precursor ink.^[^ ^64^ ^]^

The modulation of viscosity in semiconducting inks enables dynamic 3D structures. Kim et al. presented semiconductor inks of surface‐oxidated (Bi,Sb)_2_(Te,Se)_3_ nanoparticle‐based p‐ and n‐type inks with thermoelectric (TE) properties, and chalcogenidometallate (ChaM) was added as a rheological modifier (Figure 3f).^[^ ^65^ ^]^ As a result, the ink was extruded through the nozzle without bulging and formed arch‐type, cylindrical architectures (Figure 3g). Cao et al. introduced viscoelastic LiFePO_4_ particle ink with cellulose nanofiber (CNF).^[^ ^66^ ^]^ The shear‐thinning properties of CNF enabled the extrusion of LiFePO_4_ with a controlled 3D structure. All CNF‐containing inks were viscous so stuck to the bottom of the vial. In contrast, the LiFePO_4_ dispersion without CNF was fluidic (Figure 3h).

Recently, numerous studies of printing organic–inorganic hybrid materials, such as perovskites, are of huge interest for their facile processing and superior semiconducting properties. Chen et al. introduced the 3D printing of halide perovskites, with their precursor solution in dimethylformamide (DMF).^[^ ^67^ ^]^ By the femtoliter meniscus between nozzle and substrate, precursors were crystallized and form 3D structures of perovskites (Figure 3i). Glushkova et al. demonstrated a precursor ink of halide perovskite in DMF, and printed the ink for fabricating 3D X‐ray photodetector.^[^ ^68^ ^]^

Carbon‐based low‐dimensional materials including graphene and CNTs have mechanical robustness, unique electronic transport properties, high carrier mobility, and relative inertness to external environments, which make them a candidate for semiconducting materials in electronics.^[^ ^69^, ^70^, ^71^, ^72^ ^]^ An et al. printed reduced graphene oxides (rGOs) as a dispersion ink in DMF.^[^ ^73^ ^]^ To prevent the clogging of the nozzle from the rapid evaporation of DMF, ethylene glycol was added in ink for retarding the evaporation. The printed rGOs worked as a channel for TFT, and showed the mobility of 6 cm^2^ V^−1^ s^−1^ (Figure 3j). Sun et al. demonstrated the semiconducting CNT inks for transistors to operate active‐matrix display (Figure 3k).^[^ ^74^ ^]^ The transistor arrays with CNT channels showed average mobility of 0.23 ± 0.12 cm^2^ V^−1^ s^−1^. There are also efforts of printing 2D inorganic compounds such as MXenes. Zhang et al. showed the formation of printable ink including MXene (Figure 3l).^[^ ^75^ ^]^ To enhance the dispersion of these 2D materials, high polarity solvents such as ethanol, DMF, and *N*‐Methyl‐2‐pyrrolidone (NMP) were used as solvents.

### 2.3 Insulators

Electrically insulating materials have been facilitated as substrates in which various electronics are formed or as dielectric materials that constitute transistors and capacitors. Recently, 3D printing of insulating materials has been demonstrated to increase the structural diversity and integrity in electronics.

#### 2.3.1 Substrates

The substrate of the electronics must provide electrical insulation and structural support for both 2D and 3D environments. Polymers are a representative material that can be used as the substrate of electronics, and their plasticity and solution‐processing ability facilitate the use as 3D‐printable inks.

**Figure 4:**
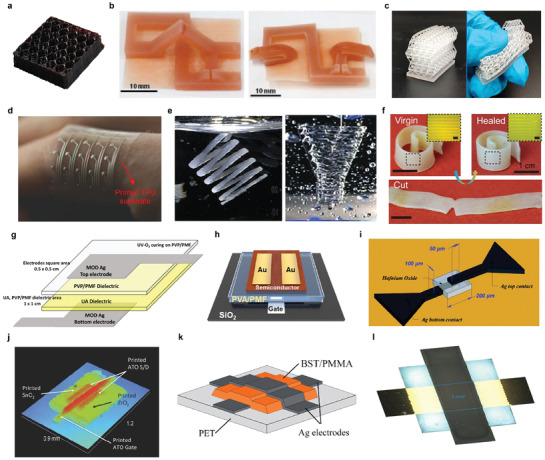
Insulators as forms of printable ink. a) Photograph of printed hexagonal honeycomb structure. b) Photographs of 3D‐printed PCL construct (left), and temporary state to enable inkjet printing on a 2D surface (right). c) Photographs of the 3D‐printed hollow lattice structure (left), and its shape deformation with finger pressing (right). d) Photograph of sensor array on printed TPU substrate. e) Photographs of FRE printed PDMS helix structure (left), and FRE printed PDMS bifurcation embedded in the Carbopol (right). f) Photographs of the self‐healing spiral structure. Inset: Optical micrograph of the surface morphology with a scale bar of 1 mm. g) Schematic illustration of the structure of the film and textile capacitors with printed PVP/PMF dielectric layer. h) Schematic illustration of the bottom‐gate‐top‐contact OFET device structure with printed PVA/PMF gate insulator layers. i) Schematic illustration of the metal‐insulator‐metal capacitor structure with HfO_2_ dielectric layer. j) Surface profile image of the TFT structure with ZrO_2_ dielectric layer. k) Schematic illustration of the printed capacitors with the BST/PMMA dielectric layers. l) Microscope image of a printed capacitor with a polymeric composite dielectric layer. a) Reproduced with permission.^[^ ^76^ ^]^ Copyright 2021, American Chemical Society. b) Reproduced with permission.^[^ ^78^ ^]^ Copyright 2015, Wiley‐VCH. c) Reproduced with permission.^[^ ^79^ ^]^ Copyright 2020, American Chemical Society. d) Reproduced with permission.^[^ ^81^ ^]^ Copyright 2017, Wiley‐VCH. e) Reproduced with permission.^[^ ^83^ ^]^ Copyright 2016, American Chemical Society. f) Reproduced with permission.^[^ ^84^ ^]^ Copyright 2018, American Chemical Society. g) Reproduced with permission.^[^ ^86^ ^]^ Copyright 2021, American Chemical Society. h) Reproduced with permission.^[^ ^87^ ^]^ Copyright 2021, Wiley‐VCH. i) Reproduced with permission.^[^ ^88^ ^]^ Copyright 2016, The Royal Society of Chemistry. j) Reproduced with permission.^[^ ^89^ ^]^ Copyright 2015, Wiley‐VCH. k) Reproduced with permission.^[^ ^90^ ^]^ Copyright 2018, Springer Nature. l) Reproduced with permission.^[^ ^91^ ^]^ Copyright 2020, American Chemical Society.

Wang et al. fabricated a polyimide (PI) ink for 3D printing, and printed the ink into honeycomb‐shaped 3D structures with excellent thermal and mechanical properties.^[^ ^76^ ^]^ First, they formed poly(amic acid) ammonium salt (PAAS) hydrogels through aqueous polymerization of biphenyl tetracarboxylicdianhydride (BPDA), phenylenediamine (PDA), and triethylamine (TEA). Second, the honeycomb structure was fabricated through inkjet printing of PAAS hydrogel. Third, thermal imidization was induced by heat treatment at the high temperature of 400 °C to fabricate BPDA‐PDA polyimide. The fabricated PI structure was thermally stable up to 590 °C, and it was confirmed that compressive strengths of 12.6–43.5 MPa were exhibited depending on the control of the density of the structure (**Figure** **4**a).

Guo et al. produced PI ink that can minimize the dimensional shrinkage of the structure that occurs after thermal imidization.^[^ ^77^ ^]^ They conducted UV‐assisted direct writing using UV‐curable hydroxyethyl methacrylate (HEMA) grafted polyamic acid (PAA) as printable ink. The printed ink was thermally imidized to form the PI structure. The presence of HEMA grafting in the main chain of PAA improved the feasibility of the PI ink by limiting the shrinkage after thermal imidization to 6%. Zarek et al. printed polycaprolactone (PCL) ink to produce memory geometries with complex shapes.^[^ ^78^ ^]^ To prepare the resin for stereolithography (SLA), PCL was linked covalently with methacrylate groups and heated to melt. The PCL structures could be used as responsive objects that enable the reversible reaction of the thermal transformation (Figure 4b).

Recently, with the development of stretchable devices, such as wearable electronics and soft robotics, the demand for flexible/stretchable substrate materials has increased. Peng et al. proposed a mechanically robust and transparent substrate by optimizing the soft segment material of polyurethane (PU).^[^ ^79^ ^]^ PU can be designed from elastomer to rigid plastic by adjusting the ratio of soft segments and hard segments present in the polymer chain. The authors synthesized polyurethane acrylate oligomers (PUAs) using three different soft segment materials. The three synthesized PUAs were used as a liquid resin in digital light processing (DLP) printing to fabricate freeform structures. Among them, the tensile strength, elongation at break, and transmittance of PPTMGA‐40, which contains poly (tetrahydrofuran) in the polymer chain, showed the highest performance with 15.7 MPa, 414.3%, and 89.4%, respectively (Figure 4c).

Patel et al. studied the mechanical properties of highly stretchable and UV curable (SUV) elastomer systems that are suitable for DLP‐based 3D printing.^[^ ^80^ ^]^ The ink that is produced was composed of epoxy aliphatic acrylate (EAA), aliphatic urethane diacrylate (AUD), and a photoinitiator. The authors evaluated the effect of the quantities of EAA and AUD on the mechanical properties, and they found that the mechanical properties increased as the amount of AUD increased. It was shown that elongation reached up to 1100% by increasing the AUD.

Valentine et al. fabricated a flexible/stretchable substrate for soft electronics using thermoplastic polyurethane (TPU) as printable ink.^[^ ^81^ ^]^ Insulating ink was prepared by dissolving TPU in *N*,*N*‐dimethylformamide (DMF), and tetrahydrofuran (THF) solvents. The TPU substrate printed through direct writing showed a low elastic modulus of 2.3 MPa and a dielectric constant of 9.1, demonstrating that it can be used as a substrate for soft electronics (Figure 4d). Similarly, Zhou et al. fabricated the substrate and sealing layer for flexible/stretchable electronics by direct writing of the silicon elastomer ink.^[^ ^82^ ^]^ The silicon elastomer ink was made by mixing fumed silica and different kinds of silicon rubber. The fabricated substrate provided structural support even in environments in which it was deformable.

Hinton et al. reported freeform reversible embedding (FRE) printing using hydrophilic carbopol support, and they fabricated a complex PDMS structure.^[^ ^83^ ^]^ When the hydrophobic PDMS prepolymer ink is printed in the carbopol gel, the carbopol acts as a Bingham plastic to the syringe tip and acts as a solid to the extruded PDMS. The FRE‐printed PDMS was not diffused into the carbopol gel during the gelation time, and it received plastic support. Through this method, the printing of complex freeform PDMS structure, such as a Helix, a helical tube, and a perfusable tube, was implemented (Figure 4e).

More recently, with the development of soft materials, there also are studies on smart functions, such as self‐healing. Kuang et al. developed a printable hybrid ink that is stretchable and has the characteristics of shape memory and self‐healing.^[^ ^84^ ^]^ The hybrid ink was produced by dissolving semicrystalline PCL in the AUD, and fumed silica nanoparticles were added as a rheology modifier. The hybrid ink was printed by using the UV‐assisted direct writing method to form various 3D structures. The printed object was stretchable up to 600% during exhibiting self‐healing and shape memory behaviors, due to the embedded semicrystalline thermoplastic (Figure 4f).

#### 2.3.2 Dielectrics

As research progresses on manufacturing electronic devices in 3D structure, there have been several attempts to 3D‐print dielectric materials for use in transistors and capacitors. Printable dielectric materials include polymers, inorganic materials, and organic/inorganic hybrid materials.^[^ ^85^ ^]^

Polymers have been studied extensively as 3D printable inks due to their low processing temperature and plasticity characteristics, despite their having a low dielectric constant value compared to inorganic materials. In particular, the brittleness of inorganic materials in flexible/soft electronics can become a limitation, but organic materials can overcome this limitation.

Kim et al. proposed an all‐inkjet‐printed textile capacitor with high chemical and mechanical durability by printing bilayer polymer dielectrics.^[^ ^86^ ^]^ The bilayer polymer was composed of UV curable urethane acrylate (UA) and poly(4‐vinyl phenol)/poly(melamine‐*co*‐formaldehyde) (PVP/PMF). The UA ink formed a flattened dielectric layer when printed on the textile substrate. As the PVP/PMF ink is printed on the UA dielectric layer, covalent bonding is formed between the UA and the PVP/PMF layer. The dielectric constant and capacitance value of the fabricated dielectric layer were 4.86 at 1 kHz and 105.07 pF cm^−2^, respectively (Figure 4g).

Jung et al. fabricated organic field‐effect transistors (OFETs) by printing the gate insulating (GI) layer with polyvinyl alcohol (PVA)‐based ink.^[^ ^87^ ^]^ The PVA‐based ink was optimized using poly(melamine‐*co*‐formaldehyde) (PMF) as a crosslinking agent, and the GI layer was formed through electrohydrodynamic inkjet printing. The effectively cross‐linked PVA chain improved the dielectric strength and showed a lower leakage current than the PVA‐only dielectric (Figure 4h).

However, due to the intrinsically low dielectric performance of polymers, research was conducted to develop inorganic dielectric materials as printable ink. Vescio et al. proposed inorganic HfO_2_ as printable ink and inkjet‐printed it as a dielectric layer of a metal‐insulator‐metal (MIM) capacitor.^[^ ^88^ ^]^ HfO_2_ printable ink was prepared by dispersing HfO_2_ NPs in a solvent that contained several components, including aliphatic compounds. The HfO_2_ dielectric layer was formed with a thickness of 400 nm and a width of 200 µm. The relative permittivity (*k*) and dielectric lost tangent (tan *δ*) of the printed HfO_2_ thin film were 12.6 and 0.0125 at l MHz, respectively, and they showed good homogeneity with the spherical‐shaped NPs that had 25‐nm diameters (Figure 4i).

Jang et al. produced a TFT by inkjet printing an inorganic ZrO_2_ dielectric layer.^[^ ^89^ ^]^ Ink for ZrO_2_ was manufactured by dissolving zirconium acetylacetonate in a mixed solution of ethanol and monoethanolamine. The poly(methyl methacrylate) (PMMA) layer was coated temporarily to control the surface energy between the printed layers, and it was removed through high‐temperature annealing. The printed ZrO_2_ dielectric layer had a dielectric constant > 20, and the leakage current was 10^−5^ A cm^−2^ at below 1 MV cm^−1^ (Figure 4j).

Recently, studies for 3D‐printable dielectric layers have been reported using hybrid ink of organic‐inorganic compounds. The hybrid ink shows the possibility of having both advantages, that is, the high‐*k* characteristics of inorganic materials and the good processability of organic materials. Mikolajek et al. developed a ceramic/polymer composite ink and printed the dielectric of the capacitor on a flexible substrate by inkjet printing.^[^ ^90^ ^]^ The composite ink was produced by mixing Ba_0.6_Sr_0.4_TiO_3_ (BST) with PMMA solution. The permittivity of the composite ink was improved compared to pure PMMA, and the dielectric constant ranged from 20 up to 55 at 1 kHz, depending on the ratio of BST and PMMA (Figure 4k). Reinheimer et al. produced a thin dielectric layer through inkjet printing of polymerizable ceramic ink.^[^ ^91^ ^]^ The hybrid ink is composed of surface‐modified BST particles, a cross‐linking agent, and a thermal radical initiator. Polymerization of ink occurs immediately on the heated substrate, and a homogeneous topography is formed. The fabricated dielectric layer revealed a high permittivity of 40 and a capacitance of 500 pF mm^−2^ when a 700 nm‐thick layer was formed on the capacitor (Figure 4l).

## 3 3D Printing Methods

### 3.1 Inkjet

In inkjet printing, liquid‐phase inks are ejected rapidly to a substrate. Currently, the most conventional printing methods are the thermal and piezoelectric inkjet methods.^[^ ^92^ ^]^ However, these two methods eject a relatively large volume of liquid‐phase ink droplets due to the surface tension of the liquid, which makes it difficult to print high‐resolution patterns as well as freestanding 3D structures. On the other hand, electrohydrodynamic jet printing and aerosol jet printing methods can form high‐resolution 3D structures by quickly evaporating the solvents in the ink by ejecting a small volume of liquid‐phase ink droplets with a high surface‐to‐volume ratio.^[^ ^93^ ^]^ Therefore, electrohydrodynamic and aerosol inkjet printings have been studied almost exclusively as a method for 3D printing.^[^ ^94^, ^95^ ^]^ In this review, the two inkjet printing methods are covered as the representative inkjet‐based 3D printing methods.

#### 3.1.1 Electrohydrodynamic Inkjet Printing

**Figure 5:**
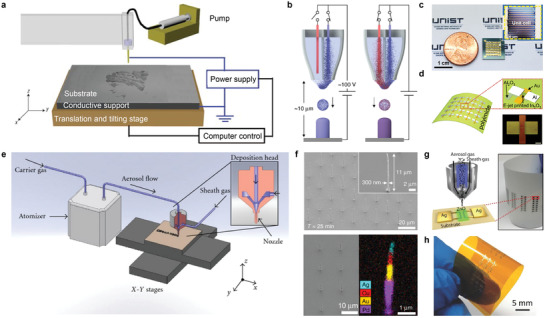
Inkjet‐based 3D printings. a) Schematic illustration of the electrohydrodynamic jet printer setup. b) Schematic illustration of the electrohydrodynamic redox printing multichannel nozzle. c) Photograph of the UHD SS‐MSCs fabricated on a chip (area = 8.0 mm × 8.2 mm, smaller than a coin). Inset shows an optical microscopy image of the unit cell in the UHD SS–MSCs. d) Schematic illustration of the e‐jet‐printed flexible In2O3 TFT array on a PI substrate and optical micrograph of a single transistor on th.e PI substrate. Scale bar, 50 µm. e) Schematic illustration of the aerosol jet printing system. f) SEM image (bottom left) and EDS map (bottom right) of nanopillars consisting of Pd (purple), Au (yellow), Cu (red) and Ag (light blue). g) Schematic illustration of aerosol jet deposition head demonstrating the aerodynamic focusing of aerosol gas stream on the device architecture of the printed photodetector on PET substrate and optical image of fully aerosol‐jet printed array of ZnO photodiodes on a mechanically flexible PET substrate. h) Photograph of flexible CNT‐TFTs and logic circuits on a Kapton film. a) Reproduced with permission.^[^ ^93^ ^]^ Copyright 2007, Nature Publishing Group. b) Reproduced with permission.^[^ ^99^ ^]^ Copyright 2019, Springer Nature. c) Reproduced with permission.^[^ ^100^ ^]^ Copyright 2020, American Association for the Advancement of Science. d) Reproduced with permission.^[^ ^63^ ^]^ Copyright 2016, The Royal Society of Chemistry. e) Reproduced under the Creative Commons Attribution License.^[^ ^101^ ^]^ Copyright 2021, Beilstein. f) Reproduced with permission.^[^ ^95^ ^]^ Copyright 2021, Springer Nature. g) Reproduced with permission.^[^ ^106^ ^]^ Copyright 2018, American Chemical Society. h) Reproduced with permission.^[^ ^107^ ^]^ Copyright 2017, Wiley‐VCH.

The electrohydrodynamic jet (e‐jet) printing method is a method in which an electric field is applied between the ink inside the nozzle and the stage where the ink is being printed (**Figure** **5**a).^[^ ^93^ ^]^ As the electric charges accumulate on the surface of the ink due to the increasing electric field, an electric repulsive force is generated between the ink particles, and a sharp meniscus is formed that is known as a Taylor cone.^[^ ^96^ ^]^ As the strength of the electric field increases and becomes sufficient enough, the electrostatic stress becomes greater than the capillary tension at the end of the liquid cone and the ink at the end of the Taylor cone is printed. In the case of the thermal or piezoelectric inkjet printing method, it is difficult to achieve high‐resolution printing because a large pressure must be applied at a level that can overcome the capillary force to eject ink through a small nozzle. However, in the case of e‐jet printing, since the ink is ejected by the charge accumulation in an electric field, the printed jet has a far narrower diameter than the used nozzle. This is because the ink droplet falls due to the strong electric field between the nozzle and the stage, and only a little lateral variation occurs so high‐resolution printing is possible.^[^ ^97^ ^]^ Also, the materials that are printed must have a certain level of mobile charge, because the ink is printed due to the electrostatic force between the mobile ions accumulated in the Taylor cone. However, in a study conducted by Jayasinghe et al., it was found to be possible to print liquids with low electrical conductivity, for example, 10^−13^ to 10^−3^ S m^−1^, which makes it possible to print various materials, including conducting materials, insulating materials, and semiconducting materials.^[^ ^98^ ^]^

Reiser et al. introduced electrohydrodynamic redox printing, which makes it possible to directly produce polycrystalline metal 3D structures without further processing after printing.^[^ ^99^ ^]^ Metal ions are generated in the solution inside the printing nozzle by using a metal anode in a liquid solvent. Then, the ion‐loaded solvent droplets are ejected onto the substrate by electrohydrodynamic forces. When these droplets contact the substrate, the transfer of electrons from the substrate reduces the ions, thereby forming a metallic deposit. Using this method, Cu patterns were printed with line widths below 100 nm. By switching the oxidative voltage between different metal electrodes inside the multi‐channel nozzle, it is possible to print either a single type of metal or an alloy of two different metals (Figure 5b).

Lee et al. produced ultra‐high areal number density solid‐state micro‐supercapacitors (UHD SS‐MSCs) by e‐jet printing nanosized‐activated, carbon‐based ink and solid gel electrolyte on a Ti/Au current collector.^[^ ^100^ ^]^ Using the e‐jet printing, interdigitated electrodes with a line width of 10 µm were printed. 36 unit cells were connected to a chip to achieve the exceptionally high areal number density of 54.9 cells cm^−2^ and an areal opening voltage of 65.9 V cm^−2^ (Figure 5c).

Kim et al. fabricated oxide semiconductor TFTs by e‐jet printing In_2_O_3_ on a PI substrate with a minimum line width of 2 µm, as shown in Figure 5d.^[^ ^63^ ^]^ Even when the TFT was peeled from the support substrate and wrapped on various curved surfaces, both the mobility and threshold voltage were observed to change less than 10% after bending, and the TFT showed excellent mechanical flexibility.

#### 3.1.2 Aerosol Inkjet Printing

The aerosol jet printing is a high‐resolution inkjet printing method that ejects ink into an aerosol with diameters of 1–5 µm using an ultrasonic or pneumatic atomizer (Figure 5e).^[^ ^101^ ^]^ After being atomized, the ink aerosol droplets move to the deposition head surrounded by the sheath gas, and they are focused in the form of a beam by the sheath gas, which is sprayed with a nozzle and printed. The distance between the nozzle and the substrate is maintained to be 2–5 mm, which makes it possible to print freeform structures on complex surfaces.^[^ ^102^, ^103^, ^104^, ^105^ ^]^

Jung et al. demonstrated 3D printing of arrays of metal nanostructures using various materials.^[^ ^95^ ^]^ When ions are directed onto a dielectric mask with an array of holes that is separated from the silicon substrate, the injected ions accumulated around the mask, and they acted as an electrostatic lens. Then, when charged aerosol particles were injected, they were printed on a silicon substrate in the form of a nanoscale jet. The printing process was conducted while moving the silicon substrate, and various 3D freeform structures were printed, such as helices, nanopillars, rings, and letters. The diameter of printed nanopillars was 85 nm. Also, a single 3D structure consisting of various types of materials can be printed through a single printing process (Figure 5f).

Gupta et al. demonstrated aerosol jet printed zinc oxide (ZnO) and its application to an ultraviolet photodetector.^[^ ^106^ ^]^ The ZnO photodetector was fabricated using a flexible polyethylene terephthalate (PET) substrate, with processing temperatures at 150 °C or less (Figure 5g).

Cao et al. presented the fabrication of all layers of hysteresis‐free carbon nanotube thin‐film transistors (CNT‐TFT) by aerosol jet printing.^[^ ^107^ ^]^ The materials used for printing were high‐purity semiconducting CNTs for the channel, Ag nanoparticle ink as the electrode, and blend of poly(vinylphenol)/poly(methyl silsesquioxane) (PVP/pMSSQ), xdi‐dcs as the gate dielectric (Figure 5h).

### 3.2 Direct Writing

**Figure 6:**
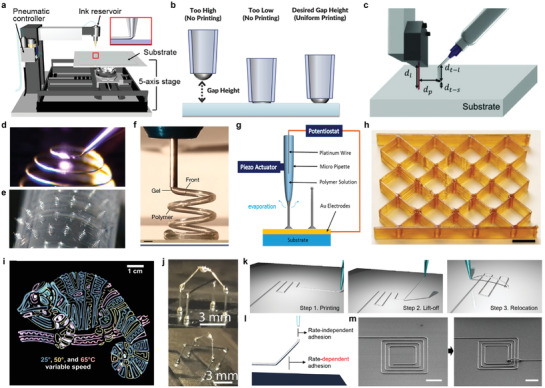
Direct writing‐based 3D printing. a) Schematic illustration of the 3D direct writing system. b) Schematic illustration of the gap height settings of direct writing. c) Schematic illustration of the major parameters associated with 4‐laser distance sensor combined liquid metal direct writing. d) Photograph of a side view showing freeform 3D printing of a metal hemispherical spiral. e) Photograph of hemispherical spiral array printed on a PET film, bent around a cylinder. f) Photograph of 3D printing of a DCPD solution that is solidified immediately after extrusion from the print head. Scale bar, 100 mm. g) Schematic illustration of the contact detectable direct writing set up. h) Photograph of the printed truss structure after sintering two times, Scale bar: 10 mm. i) Photograph of a complex photonic pattern printed in three layers at three substrate temperatures. j) Schematic illustrations of each step of the 3D reconfiguration. k) Schematic illustration of two adhesion forces during reconfiguration. l) SEM images of reconfigured square coils. The end of the inner line in the square coil (left) is lifted and reconfigured (right), Scale bars, 200 µm. m) Photograph of a complex 3D network of freeze‐printed EGaIn. a, k‐m) Reproduced with permission.^[^ ^9^ ^]^ Copyright 2019, American Association for the Advancement of Science. b) Reproduced with permission.^[^ ^110^ ^]^ Copyright 2020, Wiley‐VCH. c) Reproduced with permission.^[^ ^112^ ^]^ Copyright 2018, Wiley‐VCH. d, e) Reproduced with permission.^[^ ^28^ ^]^ Copyright 2016, National Academy of Sciences. f) Reproduced with permission.^[^ ^115^ ^]^ Copyright 2018, Springer Nature. g) Reproduced with permission.^[^ ^116^ ^]^ Copyright 2018, American Chemical Society. h) Reproduced with permission.^[^ ^117^ ^]^ Copyright 2019, Wiley‐VCH. i) Reproduced with permission.^[^ ^118^ ^]^ Copyright 2020, American Association for the Advancement of Science. j) Reproduced with permission.^[^ ^121^ ^]^ Copyright 2016, Wiley‐VCH Verlag.

Direct writing is a nozzle‐based, drop‐on‐demand 3D printing method that extrudes viscous ink by pneumatic pressure.^[^ ^108^, ^109^ ^]^ The computer‐controlled delicate control combined with a direct writing system enables the formation of complex and fine patterns with high resolution.^[^ ^110^ ^]^ **Figure** **6**a illustrates the schematic illustration of the direct writing system. The system consists of the ink reservoir which contains the ink and the nozzle (e.g., glass capillary, metal) where the extrusion of the ink is made, a moving stage, and the pneumatic pressure controller which manipulates the amount of pressure pushing the ink out of the nozzle. In the case of general 2D direct writing, it is done in the following order. First, a meniscus is formed at the tip of the nozzle by pushing the ink from the ink reservoir toward the nozzle with pneumatic pressure. Then, wetting through the interaction between the substrate and the ink is achieved by making a firm contact between the substrate and the nozzle. When sufficient wetting occurs, shear‐driven printing is performed in the form of drawing ink on the substrate by moving the nozzle or stage in the *xy*‐plane.^[^ ^111^ ^]^ The pressure applied to the ink inside the nozzle, and the distance between the substrate and the nozzle must be adjusted properly so that the ink at the tip of the nozzle and the substrate are in proper contact (Figure 6b).^[^ ^110^ ^]^ The remaining challenge is that it is difficult to maintain the proper contact when printing on an inclined or curved substrate. To deal with this challenge, Yoon et al. built a system that measures the distance between the nozzle and the substrate.^[^ ^112^ ^]^ By considering the four major distance parameters (laser sensor to the substrate (*d*_l_), laser sensor to the dispensing needle tip (*d*_t–l_), dispenser tip to the substrate (*d*_t–s_), measuring spot of the laser sensor to the dispenser tip (*d*_p_)) real‐time adjustment of the distance between the nozzle and the substrate was made for being printable in curved substrates (Figure 6c).

To direct‐write a freeform 3D structure, the ink should maintain its structure even after the ink is extruded from the nozzle and printed on the substrate. Therefore, ink capable of maintaining the freeform in the ambient air should be used. To enable the ink to maintain its as‐printed structure, various studies of the ink for direct writing, such as viscoelastic slurry colloidal ink that mixes the metallic powder into a polymer binder or organic solvent medium or polymer‐based ink with adequate viscosity are conducted.^[^ ^113^, ^114^ ^]^ Also, post‐printing processes such as sintering, or annealing are made after 3D printing. Skylar‐Scott et al. used an 808 nm wavelength IR laser for localized annealing to a colloidal form of Ag nanoparticle ink.^[^ ^28^ ^]^ The assist of the laser enabled the direct writing to a temperature‐sensitive plastic substrate such as PET. This laser‐assisted direct ink writing decreased process time by printing the 20 µm width 3D line and annealing in a single step, enabling the formation of high conductivity freeform 3D metallic architecture without a high‐temperature annealing process (Figure 6d). Figure 6e shows the hemispherical spiral array printed on a PET film then bent around a cylinder. Robertson et al. mixed phosphite‐inhibited dicyclopentadiene (DCPD) and second‐generation Grubbs’ catalyst to form a polymer ink that exists in a liquid phase and slowly transforms into a highly viscous elastomeric gel in the air.^[^ ^115^ ^]^ The alignment of the velocity of the print‐head and polymer curing velocity enabled the maintenance of the 3D structures after printing, as the liquid polymer ink cures immediately after extruding out of the nozzle (Figure 6f). Yuk et al. direct‐wrote a PEDOT:PSS hydrogel ink to a substrate.^[^ ^44^ ^]^ To remove the DMSO and the deionized water to form a percolation between PEDOT‐rich crystal‐line domain and PEDOT:PSS nanofibril, drying at 60 °C for 24 h, and dry annealing three times in 130 °C for 30 min was performed. Zhang et al. showed a unique direct writing method by inserting the platinum (Pt) wire with 0.125 mm diameter as a counter electrode inside the micropipette nozzle as the potential difference between the substrate and the counter electrode induces a difference in the drain current, indicating whether the substrate and the nozzle are in contact (Figure 6g).^[^ ^116^ ^]^ In this way, the 3D pillar structure of PEDOT:PSS with various sizes was printed on an Au electrode and the printing condition of each structure was optimized. This method produced pillar structures that had an extremely high aspect ratio with 7 µm in diameter and 5000 µm in height.

Direct writing has the advantage of being able to easily print freeform structures with multi‐materials regardless of the type of material if only the rheological properties are matched. In general, multiple nozzles are used for direct writing multi‐materials, adding complexity to the motion control. Chen et al. direct wrote a hybrid nanocomposite ink consisted of a photocurable, thermally curable resin and silica nanoparticles for viscosity control by a single nozzle.^[^ ^117^ ^]^ After printing each layer, a 3D structure was formed by photocuring using a photomask, and the as‐printed structure was finally thermally cured in the oven to add stability (Figure 6h). Patel et al. synthesized the poly(dimethylsiloxane)‐block‐poly(lactic acid) bottlebrush block copolymer (BBCP) for the ink.^[^ ^118^ ^]^ By mixing BBCP with volatile solvent tetrahydrofuran, it could be direct‐written to the substrate. With maintaining moderate spatial resolution, the BBCP was printed to a heated Si substrate with a speed range of 15–360 mm min^−1^ forming a complex photonic pattern (Figure 6i).

Liquid metal has advantageous rheological conditions for the ink of direct writing because it can maintain the freeform 3D structure after the printing due to the oxide layer that formed on the surface.^[^ ^119^ ^]^ Since liquid metal has high electrical conductivity, research of forming 3D functional structures through direct writing of liquid metal ink has been conducted actively.^[^ ^120^ ^]^

Gannarapu et al. developed a freeze printing method by dispensing EGaIn in a cold substrate at a temperature lower than the melting point of EGaIn.^[^ ^121^ ^]^ This method was able to maintain the complex 3D structure without any supporting material due to the enhancement of the mechanical properties of the freeze‐front. The enhanced structural stability allowed direct writing with the changed printing angle to form a bridge‐type structure, which is shown in Figure 6j. Narrow metallic filaments can be advantageous for maintaining free‐standing 3D structures of liquid metal by increasing the relative surface area of robust oxide skin to fluidic volume. However, commercially available 3D printing techniques used to form the conductive 3D patterns possesses resolutions limited to a scale above ≈30 µm, which is too large for use in electronics.

Park et al. developed a high‐resolution 3D printing of EGaIn through a lift‐off process (Figure 6k).^[^ ^9^ ^]^ Using a narrow‐diameter nozzle made by pulled glass capillary, a minimum line width of 1.9 µm was printed. When printed at a speed ranging from 0.001 to 0.1 mm s^−1^, the adhesive energy of the EGaIn‐substrate interface is smaller than the fracture energy of the oxide skin (Figure 6l). Figure 6m shows the relocated square coil showing breakthrough of the limitations of the 2D interconnects.

Direct writing has the advantage of being able to produce delicate and complex 3D printing, but several challenges must be overcome. First, because printing is directly printed on the substrate, the printing is restricted depending on the condition of the surface and the relationship between the ink and the substrate. In the case of liquid metal, it can be fully wetted at a flat surface. But when it comes to a rough surface in micro and nanounits, numerous air pockets are formed between the ink and the substrate, resulting in a solid‐liquid‐air system and a decrease in surface adhesion.^[^ ^122^ ^]^

### 3.3 Photocuring

The photocuring methods create 3D structures by exposing the liquid monomer or oligomer resin to light that has a specific wavelength. The photocuring method is used in research requiring high‐resolution 3D printing and freeform electronics beyond the conventional framework because the resolution of the cured polymer can be freely controlled by adjusting the amount of photon irradiated with light and focusing. The first record of the use of photocuring in 3D printing was in 1981 by Hideo Kodama, who continuously solidified thin layers of a polymer to make plastic parts, and a paper was published explaining three basic techniques.^[^ ^123^ ^]^ Subsequently, in 1986, the stereolithography (SLA) method was patented by Chuck Hull using the light method of 3D printing technology that had matured rapidly in the early stages.^[^ ^124^ ^]^ Generally, UV light (short wavelength < 400 nm) is used for photocuring, but a range of other wavelengths of light may be selected depending on the kind of initiator that is included in the resin. UV light has a shallow penetration depth of about 100 µm from the surface of the resin, and the speed of 3D printing is slow. In addition, when a polymer is exposed to high‐energy UV light for a long time, toxic substances that can affect the environment and living organisms adversely can be produced by the occurrence of side reactions. In particular, the use of UV light in bioprinting causes a risk of cell damage. Therefore, as a solution, studies have been conducted to develop photocuring methods that use visible light and near‐infrared (NIR) light, both of which have lower energy than UV.

**Figure 7:**
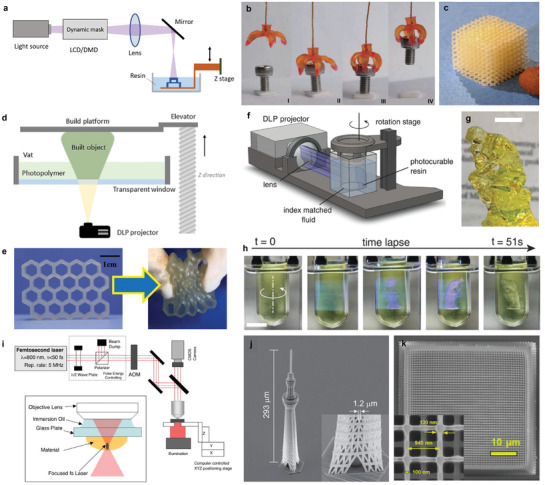
Photocuring‐based 3D printing. a) Schematic illustration of the stereolithography (SLA). b) Photographs of actuator fabricated using the SLA method. c) Photographs of gyroid architecture for use as a tissue scaffold. d) Schematic illustration of the DLP. e) Photographs of the printed honeycomb‐shaped sample with the photopolymer. f) Schematic illustration volumetric DLP printing methods. g) “Rodin's The Thinker” 3D‐printed by DLP. Scale bar, 10 mm. h) Photograph of sequential view of the build volume during the print. Scale bar, 10 mm. i) Schematic illustration of the experimental setup for TPP. j) SEM of “Tokyo Skytree” by using TPP. k) SEM of cell scaffolds array. Scale bar, 25 µm. a) Reproduced with permission.^[^ ^125^ ^]^ Copyright 2020, MDPI. b) Reproduced with permission.^[^ ^126^ ^]^ Copyright 2019, American Chemical Society. c) Reproduced with permission.^[^ ^127^ ^]^ Copyright 2009, Elsevier. d) Reproduced with permission.^[^ ^128^ ^]^ Copyright 2018, Wiley‐VCH. e) Reproduced with permission.^[^ ^129^ ^]^ Copyright 2019, American Chemical Society. f) Reproduced with permission.^[^ ^132^ ^]^ Copyright 2019, The American Association for the Advancement of Science. g) Reproduced with permission.^[^ ^132^ ^]^ Copyright 2019, The American Association for the Advancement of Science. h) Reproduced with permission.^[^ ^132^ ^]^ Copyright 2019, The American Association for the Advancement of Science. i) Reproduced with permission.^[^ ^139^ ^]^ Copyright 2012, Laser Institute of America. j) Reproduced with permission.^[^ ^140^ ^]^ Copyright 2013, Springer Nature. k) Reproduced with permission.^[^ ^141^ ^]^ Copyright 2021, Elsevier.

**Figure** **7**a shows a schematic illustration of SLA.^[^ ^125^ ^]^ The SLA method uses specialized software to cut the 3D model into thin layers, and then irradiate the surface of the liquid polymer by laser, along the shape of the layer to cure the polymer. Then, the substrate is moved one layer in the vertical direction, and the next layer is created by irradiating the previous layer with the laser. In this way, the object is completed by the beam that is directed layer by layer onto the liquid. A support structure is needed to express the protrusion and space of the 3D model, and the supports must be trimmed after the printed model is taken out of the vat. Photopolymerization‐based 3D printing technology is divided into a top‐down method and a bottom‐up method depending on where the beam is scanned. The top‐down approach places the source of the beam above the vat and irradiates it with the laser, so the hardened part is gradually is immersed in the vat. Conversely, the bottom‐up method places the source under the vat, the platform is raised gradually into the air, and the final product is obtained in an upside‐down form. Recently, with the development of projection micro‐stereolithography (μSLA)‐based 3D printing, a high resolution of 0.6 µm has been achieved, one step closer to application to freeform and complex structures. Figure 7b,c show the actuator fabricated using the SLA method and the gyroid architecture for use as a tissue scaffold, respectively.^[^ ^126^, ^127^ ^]^

Figure 7d shows a schematic illustration for DLP that was invented by Larry Hornbeck in 1987. DLP is similar to SLA, but it has some important improvements.^[^ ^128^ ^]^ While SLA cures the polymer by gradually illuminating one layer along the line, DLP printing is faster than that of SLA because the polymer can be cured by exposing the entire layer to light at the same time. DLP uses a top‐down method, that is, light from the projector is transmitted to the polymer through the transparent bottom of the vat to initiate photopolymerization. The projector irradiates the beam by using a digital screen that consists of square pixels, and each pixel creates a mask on the surface of the polymer layer by controlling the micro‐sized mirror grid with a computer. The cured rectangular shape of the polymer layer has the same area as a pixel, and it is called a voxel. The DLP method has the advantage that the resolution can be adjusted freely, so the position error in the *xy*‐plane is small because the pixel size can be adjusted. The printed line widths are about 50 µm in all axes, depending on the projector's resolution which was generally between 35 and 100 µm. Due to these advantages, DLP typically is used for ceramic materials, and it is currently the most used technology because it has high density and hardness which provide a high‐quality surface. However, the printed results generally have poor mechanical properties, and there is a risk of their becoming brittle and potentially cracking after some time passes. Therefore, as a countermeasure, research is being conducted on methods for 3D printing elastomers and healing printed polymers (Figure 7e).^[^ ^129^, ^130^, ^131^ ^]^ Figure 7f shows another type of DLP printing that has been reported, that is, by the use of an irradiating light while rotating a vat.^[^ ^132^ ^]^ In this method, the polymer does not have to be transparent, and the printing speed is much faster (completed within 1 min) than the conventional method (Figure 7g,h).

Continuous liquid interface printing (CLIP) is an advanced method from DLP, and it was developed by Joseph Desimone in 2015.^[^ ^133^, ^134^, ^135^, ^136^ ^]^ The bottom plate of the vat in CLIP uses a transparent Teflon window, and it can transmit UV light as well as oxygen. CLIP is based on a bottom‐up method in which the printing object rises slowly enough to maintain contact with the polymer resin. The resin that is located just above the oxygen‐permeable window is not cured because a dead zone is created by the oxygen, so it can maintain a continuous liquid interface and prevent the resin from adhering to the window. The curing of the resin begins when it is no longer affected by oxygen as the material gradually moves upward from the dead zone, and this process can provide fast and continuous printing. Since CLIP does not require a recoating process, 3D printing speed is >100× faster than that of conventional DLP, which is expected to contribute to improving the production speed of freeform electronics. Also, compared to the SLA and DLP methods which have non‐uniform strength on the printing direction(vertical or horizontal) and have low mechanical strength due to weak interlayer bonding, CLIP has superior, isotropic mechanical strength.

Maruo et al. reported two‐photon polymerization (TPP) for 3D printing.^[^ ^137^, ^138^ ^]^ Unlike previous UV light‐based techniques, TPP 3D printing uses a laser source, which produces pulses in the visible and near‐infrared range in femtoseconds (Figure 7i).^[^ ^139^ ^]^ TPP can polymerize nanometer‐scale (≈100 nm) small objects, and it is applicable to the production of parts with complex and fine shapes (Figure 7j,k).^[^ ^140^, ^141^ ^]^ Since most polymers hardly absorb near‐infrared rays, if the TPP method is used, the laser can penetrate deeply into the vat and cause polymerization at the center of the resin. However, TPP can use only transparent polymer materials to focus the two lasers, which means the opaque polymers used in SLA and DLP processes cannot be used for TPP. Unlike other 3D printing techniques that use UV light, TPP enabled in‐vivo usage and made it possible to fabricate more complex freeform structures.

The viscosity of photopolymers that are used for photocuring‐based 3D printing should be low (generally <5 Pa s), because, if the viscosity is too high, it takes a long time to print, and the printed object may be distorted.^[^ ^142^, ^143^ ^]^ Also, the printing polymer must have stability and a fast curing rate when exposed to a laser. And the polymer must create a network with high mechanical strength that is sufficient to withstand the printing process. The characteristics of various 3D printing methods are summarized in **Table** **1**.

**Table 1:** Characteristics of diverse 3D printing methods for freeform electronics

Printing methods		Advantages	Disadvantages	Materials	Printing resolution	Applications
Inkjet printing	E‐jet	‐ High resolution ‐ Variety of materials	‐ Nozzle clogging problem ‐ Highly dependent on substrate conductivity ‐ Low yielding rate	‐ Conducting material^[^ ^100^, ^150^, ^204^, ^205^ ^]^ ‐ Semiconducting material^[^ ^63^, ^206^, ^207^, ^208^ ^]^ ‐ Insulating material^[^ ^209^, ^210^, ^211^ ^]^	>50 nm^[^ ^212^ ^]^	‐ Supercapacitor^[^ ^100^ ^]^ ‐ Thin‐film transistor^[^ ^63^ ^]^ ‐ Interconnect^[^ ^150^ ^]^
	Aerosol jet	‐ High resolution ‐ Variety of materials ‐ Complex patterns printable on nonplanar surfaces	‐ Substantial amount of overspray ‐ Supplying system required ‐ Coffee‐ring effect	‐ Conducting material^[^ ^95^, ^107^, ^149^, ^213^, ^214^ ^]^ ‐ Semiconducting material^[^ ^106^, ^107^, ^215^, ^216^ ^]^ ‐ Insulating material^[^ ^217^, ^218^ ^]^	>85 nm^[^ ^95^ ^]^	‐ Photodetector^[^ ^106^ ^]^ ‐ Thin‐film transistor^[^ ^107^ ^]^ ‐ Interconnect^[^ ^149^ ^]^
Direct writing		‐ High resolution ‐ Complex patterns printable ‐ Fast printing speed ‐ One step process	‐ Highly dependent on the rheology between ink and substrate ‐ Low scalability ‐ Nozzle processing required	‐ Viscous ink^[^ ^54^, ^108^ ^]^ ‐ Liquid metal^[^ ^9^, ^56^ ^]^	>630 nm^[^ ^195^ ^]^	‐ Interconnect^[^ ^9^, ^56^ ^]^ ‐ Battery^[^ ^159^ ^]^ ‐ Antenna^[^ ^170^, ^171^, ^178^ ^]^
Photocuring	SLA	‐ Easy set‐up conditions ‐ Large scale printable	‐ Volume shrinkage after printing ‐ Slow printing speed	‐ Photocurable resin ‐ Ceramic resin	>0.6 µm^[^ ^219^ ^]^	‐ Tissue scaffold^[^ ^127^ ^]^
	DLP	‐ High precision ‐ Fast printing speed	‐ Volume shrinkage after printing ‐ Small scale printable only ‐ High cost	‐ Photocurable resin ‐ Ceramic slurry	>50 µm^[^ ^220^ ^]^	‐ Dental field^[^ ^221^ ^]^ ‐ Jewelry casting^[^ ^222^ ^]^
	CLIP	‐ Fast printing speed ‐ High mechanical strength	‐ Coarse resolution ‐ High cost	‐ Low viscosity photocurable resin	>100 µm^[^ ^133^ ^]^	‐ Point‐of‐care service^[^ ^133^ ^]^ ‐ High strength‐to‐weight‐ratio^[^ ^133^ ^]^
	TPP	‐ High resolution ‐ In‐vivo usable	‐ Transparent materials printable only ‐ Slow printing speed	‐ Transparent photocurable resin	∼100 nm^[^ ^223^ ^]^	‐ In‐vivo biomedical applications^[^ ^138^, ^224^ ^]^

## 4 3D Printing of Device Components for Freeform Electronics

### 4.1 Interconnects

The interconnects, which electrically connect electrodes and components in a circuit, are essential components in electronic devices. Especially, as the devices become smaller and the degree of integration increases, the structures of devices have been developed into a high‐resolution 3D form, which resulted in the development of 3D printing technology to form conformal 3D interconnects and freestanding 3D interconnects.

**Figure 8:**
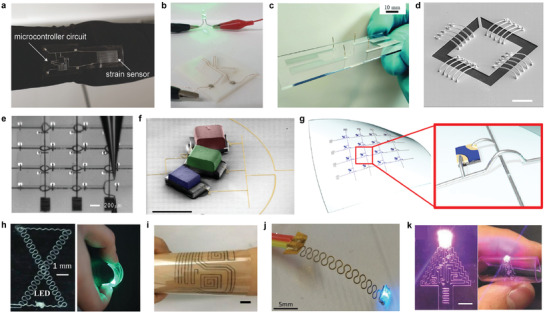
3D‐printed interconnects. a) Image of textile‐mounted printed strain sensor and microcontroller unit. b) Photograph of 3D Cu circuit formed by 3D printing and 3DLW process. c) Four printed and sintered silver pillars using silver nanoparticle ink. d) SEM image of 20 electrodeposited interconnects. Scale bar, 10 µm. e) Optical image of patterning of silver interconnects on a gallium arsenide‐based, 4‐by‐4 LED chip array. f) Colorized SEM image of three LED pixels and EGaIn interconnects. Scale bar, 1 mm. g) Schematic illustration of the microLED array with reconfigured 3D interconnects. h) Photographs of e‐jet printed two‐layer circuit that connected a battery to light a LED. i) Photograph of circuits in a flexible substrate (PET) with the GO/MWCNT hybrid ink. Scale bar, 1 cm. j) Photograph of a two‐terminal LED powered through a dual‐path FSCW. k) Photograph of a e‐jet printed conductor on PDMS with Christmas tree pattern that connected a battery to light a LED. Scale bar, 500 µm. a) Reproduced with permission.^[^ ^81^ ^]^ Copyright 2017, Wiley‐VCH. b) Reproduced with permission.^[^ ^144^ ^]^ Copyright 2020, Wiley‐VCH. c) Reproduced with permission.^[^ ^145^ ^]^ Copyright 2019, Wiley‐VCH. d) Reproduced with permission.^[^ ^8^ ^]^ Copyright 2010, The American Association for the Advancement of Science. e) Reproduced with permission.^[^ ^146^ ^]^ Copyright 2009, The American Association for the Advancement of Science. f,g) Reproduced with permission.^[^ ^9^ ^]^ Copyright 2021, American Association for the Advancement of Science. h) Reproduced with permission.^[^ ^147^ ^]^ Copyright 2021, Wiley‐VCH. i) Reproduced with permission.^[^ ^148^ ^]^ Copyright 2018, American Chemical Society. j) Reproduced with permission.^[^ ^149^ ^]^ Copyright 2019, Wiley‐VCH. k) Reproduced with permission.^[^ ^150^ ^]^ Copyright 2017, Wiley‐VCH.

Valentine et al. demonstrated a hybrid 3D printing method of electrical interconnects through the direct writing method.^[^ ^81^ ^]^ A microcontroller device including a strain sensor and a large‐area soft sensor array was fabricated (**Figure** **8**a). TPU was used as the insulating matrix and the conductive electrode ink was produced by adding silver flakes to the pure TPU ink. They printed TPU as the insulating matrix, and then printed silver flake ink for 3D conductive interconnects or strain sensors.

Jo et al. demonstrated 3D printing of a viscous fluid comprised of microsized Cu flakes and surface oxide‐free Cu nanoparticles combined with a 3D surface‐conformal laser writing technique.^[^ ^144^ ^]^ Cu‐based mixed particle paste ink (MP‐paste) was printed on top of arbitrarily shaped polymeric structures with various degrees of slopes. Even when the MP‐paste was printed on these slopes, it did not flow by the gravitational force, which makes it possible to print form‐factor free electrical circuits using the MP‐paste. The resistance was measured to be 1.7 Ω cm^−1^ and was used as an interconnect (Figure 8b).

Sowade et al. formed pillar‐shaped vertical interconnects through inkjet‐printing, using silver nanoparticle ink as shown in Figure 8c.^[^ ^145^ ^]^ The maximum height of the printed pillar structure had a 10‐mm height with a 120‐µm diameter, which had an aspect ratio of 50:1. Each end of the vertical pillars was connected to a light‐emitting diode (LED) and a screen‐printed battery. After printing the silver pillars, a UV‐cured polymer block was printed around the pillars to prove the functionality of the 3D interconnects. The measured resistivity of the silver interconnects increased from 0.34 ± 0.18 Ω cm before printing the polymer body, to 0.48 ± 0.22 Ω cm after printing the polymer body.

Hu et al. demonstrated an automated direct‐writing wire‐bond technology that exploits meniscus‐confined 3D electrodeposition in an ambient air environment.^[^ ^8^ ^]^ As the micropipette containing the electrolyte approached a conductive substrate, a meniscus was formed between the nozzle and the substrate. When an appropriate electrical potential was applied between the electrolyte and the substrate, 3D structure could be formed through electrodeposition. If the withdrawal speed of the micropipette and the growth rate of the deposit was synchronized, the stable formation of the meniscus was maintained, sustaining the growth of the interconnect. Figure 8d shows the result of this electrodeposition‐based wire bonding of Cu wires, which had a diameter of ≈800 nm.

Ahn et al. demonstrated an omnidirectional printing of freeform silver microelectrodes with minimum widths of ≈2 µm using the direct ink writing method.^[^ ^146^ ^]^ Interconnects for the gallium arsenide‐based LED array is shown in Figure 8e. The out‐of‐plane printing enabled the electrodes to form spanning arches.

Park et al. presented a method of high‐resolution direct printing of liquid metal with a minimum line width of 1.9 µm and their reconfiguration into the 3D structure.^[^ ^9^ ^]^ As shown in Figure 8f, when the anodes of the three LEDs were all connected through EGaIn printing, the LED light was enlightened by reconfiguring and connecting the freestanding interconnect to the cathode of one of the three LEDs. Also, interconnects were formed for an array of microLEDs by this reconfigurable printing method (Figure 8g). Unlike the general method of forming interconnects of multiple layers and processes, freestanding 3D interconnects through reconfiguration can form interconnects with diverse structures in a single layer and form much fewer interconnects than conventional methods.

Ren et al. used low‐melting‐point metal ink, Field's Metal (32.5% Bismuth, 51% Indium, 16.5% Tin), to print vertical interconnect accesses (VIAs) using the electrohydrodynamic printing technology to form vertical interconnects.^[^ ^147^ ^]^ A two‐layer circuit overlapped over the same area was interconnected by the printed VIAs and was connected to a battery to light a LED as shown in (Figure 8h). The circuit remained conductive even when the device was bent, stretched, and twisted. Tang et al. printed graphene aerogels (GA) and graphene‐based mixed‐dimensional hybrid aerogels (G‐MDHA) by developing hybrid inks and using a modified 3D printing system.^[^ ^148^ ^]^ Various forms of structures were printed including circuits on a flexible PET substrate using graphene oxide and multi‐walled CNTs (Figure 8i).

Jing et al. printed silver nanoparticulate ink and polyimide (PI) ink using the aerosol jet printing method to build fully freestanding stretchable conductive wires (FSCW).^[^ ^149^ ^]^ PI was printed on the spin‐coated PVA layer on top of the glass substrate. Then, the mixture of PI ink and Ag ink, achieved by combining the aerosol flow of the individual inks, was printed with the same pattern over the previously printed PI pattern. After curing the printed pattern, it was placed into water to dissolve the PVA layer, lifting the FSCW. A dual‐path FSCW was demonstrated and was used as an interconnect of a battery with a LED as shown in (Figure 8j). The voltage and current measured over the LED were not affected by up to 100% extension.

Han et al. e‐jet printed a conductive path with great flexibility and stretchability by printing a low‐melting‐point metal alloy with sub 50 µm resolution.^[^ ^150^ ^]^ They used this to print a tree‐shaped conductor that connects the battery and LED as shown in Figure 8k, and showed that the conductor remained conductive even at the bending state.

### 4.2 Batteries

Batteries are essential for operating electronic devices. Currently, the shapes of most batteries are standardized in a circular or rectangular structure optimized for coin cell or pouch cell production. However, in the case of small electronic devices that have confined scales and designs, the battery occupies most of the volume, so the technology for freely designing the shape and dimension of the battery is required for efficient utilization of space. The battery is expected to become smaller and has more diverse freeform structures for future devices, such as wearable devices, bio‐implantable devices, and small robots.^[^ ^151^, ^152^, ^153^, ^154^ ^]^ To solve this problem, research has been reported on transforming the existing 2D structure into a high‐resolution 3D structure to design the battery. In this case, the battery is smaller, but the energy density can be increased due to the increased volumetric usage of the electrodes. The advantages of 3D structures over conventional 2D structures have been demonstrated through calculations on 3D interdigitated electrode arrays. The energy capacity of a 3D design always is lower than that of a 2D design for the same total volume. However, in the 3D structure, the diffusion path is shorter than that of the 2D structure during ion transport and the resistance is smaller so that a battery with high energy density can be obtained as a result.

Batteries are made by assembling the four main components, that is, the anode, cathode, electrolyte, and separator. Since the productivity of the battery depends on the assembly process, the improvement of the assembly process is essential to increase productivity. 3D printing technology can lower the limitations of battery design for freeform electronics, and it can minimize the assembly process and dramatically increase productivity because the cost of the process is relatively low due to the use of less material and the short manufacturing time.^[^ ^104^, ^155^ ^]^ When manufacturing a battery, the thickness of the electrodes and the distance between the electrodes are kept as small as possible to minimize power losses due to the slow movement of ions. Therefore, a high‐resolution 3d printing method is essential for manufacturing a battery having a small distance interval. However, if the thickness of the electrode is reduced too much, the energy capacity and operating time become smaller.^[^ ^156^ ^]^ Therefore, it is important to adjust the balance between the two when designing the battery.^[^ ^157^ ^]^

**Figure 9:**
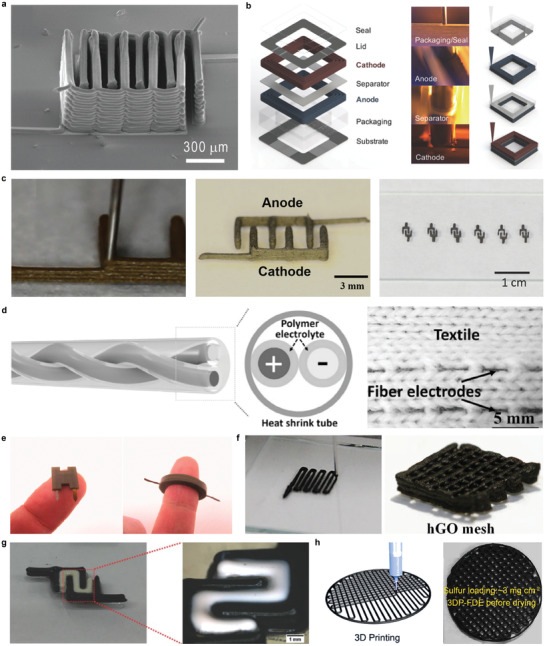
3D‐printed batteries. a) SEM of printed and annealed 16‐layer interdigitated LTO‐LFP electrode architectures. b) Schematic illustration of fully 3D‐printed Li‐ion square cell battery. c) Photograph of showing the multilayered structure being printed(left), the interdigitated electrodes(middle), and 3D‐printed electrode arrays(right). d) Schematic illustraion of the design concept for all‐fiber flexible LIBs(left) and a photograph of integration of fiber electrodes into textile fabrics(right). e) Photographs of assembled Zn‐PANI batteries in ring‐, H‐, and cylindrical shapes. f) Photograph of showing the process of printing complex 3D architectures line‐by‐line(left) and printed hGO mesh (0.8 mm line spacing). g) Photograph of the 3D‐printed interdigitated full cell battery composed of LTO/INK‐2/LFP. h) Schematic illustration of the 3D‐printing process for S/BP 2000 thick cathodes(left) and photograph of fabricated with a grid structure of 3DP‐FDE(right). a,b) Reproduced with permission.^[^ ^5^ ^]^ Copyright 2018, Wiley‐VCH. c) Reproduced with permission.^[^ ^159^ ^]^ Copyright 2016, Wiley ‐VCH. d) Reproduced with permission.^[^ ^160^ ^]^ Copyright 2017, Wiley‐VCH. e) Reproduced with permission.^[^ ^161^ ^]^ Copyright 2018, American Chemical Society. f) Reproduced with permission.^[^ ^162^ ^]^ Copyright 2018, Wiley‐VCH. g) Reproduced with permission.^[^ ^164^ ^]^ Copyright 2018, Wiley‐VCH. h) Reproduced with permission.^[^ ^165^ ^]^ Copyright 2018, Elsevier.

Although 3D printing technology is very simple, optimization of the composition and rheology of the ink is very important to prevent the adhesion of printed ink and clogging of the nozzles.^[^ ^158^ ^]^ Sun et. al. selected the Li_4_Ti_5_O_12_ (LTO) and LiFePO_4_ (LFP) as active materials for the positive and negative electrodes, respectively, and printed them on a pre‐patterned interdigitated gold current collector on a glass substrate to show a battery being produced (**Figure** **9**a).^[^ ^5^ ^]^ The anode is printed with LTO functional ink, and the cathode is printed with LFP functional ink, and then annealed at 600 °C for 2 h in the Ar gas atmosphere. After that, PMMA (Polymethyl methacrylate) was cut thinly with a laser, placed around the battery, filled with a standard liquid electrolyte (1 M LiClO_4_ 1:1 EC:DMC), and sealed with cured PDMS for packaging. Compared to a conventional battery, the battery had a high energy density in terms of area and volume due to the short diffusion distance. Wei et al. also used LTO and LFP as electrodes, while modifying the current collector to glassy carbon to make and printing inks for packaging and separator, composed of UV‐curable ceramic‐filled polymer composites (Figure 9b).^[^ ^5^ ^]^ As a result, the battery had four times the performance by using a UV curing package and increasing the area capacity by ten times compared to the previous result.^[^ ^5^ ^]^

Fu et al. fabricated all components of a lithium‐ion battery using direct writing‐based 3D printing (Figure 9c).^[^ ^159^ ^]^ They developed a water‐based graphene oxide‐based electrode composite ink and solid electrolyte ink composed of a highly concentrated graphene oxide sheet with optimal viscoelasticity and viscosity and positive and negative active materials to make all 3D‐printed batteries. LFP and LTO were selected as cathode and anode materials, respectively. To create a high mass load per unit area, microfilaments were extruded directly from the nozzle, and then the interdigitated pattern was printed layer by layer using a preprogrammed printing routine. The graphene oxide flakes were aligned along the extruded direction due to the shear stress induced by the nozzle, thereby improving the electrical conductivity of the entire electrode. In addition, the graphene oxide flakes had a large surface area due to their intrinsic porous structure, so it was possible to accommodate LFP and LTO nanoparticles and electrolytes. Wang et al. developed fibrous LIBs made by twisting printed LFP and LTO fibers together with PVDF and CNT dissolved in gel polyelectrolyte NMP (Figure 9d).^[^ ^160^ ^]^ The fiber‐type LIB produced by the direct‐write printing method exhibited the applicability to freeform electronics with high specific discharge capacity and excellent flexibility. Due to the porous structure of the poly(vinylidene fluoride‐*co*‐hexafluoropropylene) (PVDF‐*co*‐HFP) coating layer, the printed electrode absorbs sufficient liquid electrolyte and induces rapid ion transport. In addition, the interconnected CNTs facilitate the transport of electrons, resulting in a battery with excellent performance.

Kim et al. developed a miniature zinc‐ion battery using 3D printing.^[^ ^161^ ^]^ They increased the freedom of battery design dramatically by using 3D printing, and they made the batteries with the desired freeform structure such as ring shapes and capital letter shapes of H and U. The 3D‐printed ring is a water‐based Zn‐ion battery that uses zinc ions (Zn^2+^) as charge carriers instead of lithium ions (Figure 9e). This system uses water as part of the electrolyte, compared to existing lithium secondary batteries that use highly flammable organic solvents as the electrolyte. Therefore, it is safe and more stable to atmospheric moisture and oxygen than organic solvent‐based batteries that are affected by moisture and oxygen. In particular, unlike organic solvents, water has an advantage for battery packaging because water does not melt 3D‐printed plastic packaging. Lacey et al. synthesized graphene oxide by direct oxidation of commercial graphene powder and direct wrote the graphene oxide ink with a 3D hierarchical nanoporous structure.^[^ ^162^ ^]^ Liquid oxidation through the simplified Hummer's method made graphene oxide hydrophilic, so it was well dispersed without the use of binders or other additives, and stable 3D printing was possible due to its viscoelastic behavior (Figure 9f). Compared with 2D filtration films, the 3D‐printed mesh structure improved active site utilization and ion transport capacity. McOwen et al. developed a multi‐solid electrolyte ink using Li_7_La_3_Zr_2_O_12_ (LLZ) garnet for application in 3D printing.^[^ ^163^ ^]^ The ink was printed on a micrometer scale by direct writing, and a variety of freeform structures was created, from creating a solid electrolyte film conformally sintered to 5–10 µm on the substrate surface to a self‐supporting structure. Cheng et al. fabricated batteries by the direct writing method using a high‐temperature extrusion robot by using polymer‐based inks on electrodes (Figure 9g).^[^ ^164^ ^]^ The PVDF‐co‐HFP‐based ink filler containing nano TiO_2_ exhibited high viscosity and very good wettability with the electrode substrate. The hybrid solid electrolyte produced by high‐temperature 3D printing was composed of a solid polymer matrix and an ion‐liquid electrolyte. The electrolyte was printed directly on the surface of the electrode, and the dense layer between the porous polymer electrolyte and the electrode reduced the interfacial resistance during high‐temperature printing. The battery with a 3D‐printed electrolyte had a higher charge/discharge capacity value and better performance compared to using the conventional solution method. Gao et al. developed a freeze‐dried sulfur/carbon composite electrode (3DP‐FDE) for 3D printing using additive manufacturing based on low‐cost commercial BP‐2000 carbon material for high‐energy‐density and high‐power Li‐S batteries (Figure 9h).^[^ ^165^ ^]^ Electrons can be transported along the 3DP‐FDE, shortening the electron transport distance, and the micropores between the fibers are advantageous for electrolyte acceptance and transport of Li^+^ ions on a microscale.

Although 3D printing has a huge potential as a next‐generation battery manufacturing technology, there are still some problems to be solved to apply 3D printing for batteries. First, the variety of 3D‐printable materials for batteries are confined. Most of the active materials applied in energy‐related applications are inorganic and some additives must be added to the ink to increase the ink viscosity. However, these additives can transmute the properties of the active material and affect the final performance of the device. In addition, the 3D‐printed electrode generally has poor mechanical properties. Therefore, additional research is needed on materials with high viscosity, small size particles, and excellent mechanical properties for application to 3D printing technology.

### 4.3 Antennas

The antenna is the most essential element in a wireless communication system of electronics because it can transmit and receive radiofrequency (RF) signals.^[^ ^166^ ^]^ Radiation characteristics, gain, and transmittance efficiency of the antenna are greatly affected by the dimensions and shape of the antenna. With the recent development of wireless communication, the demand for antennas of various sizes, shapes, and materials is increasing.

**Figure 10:**
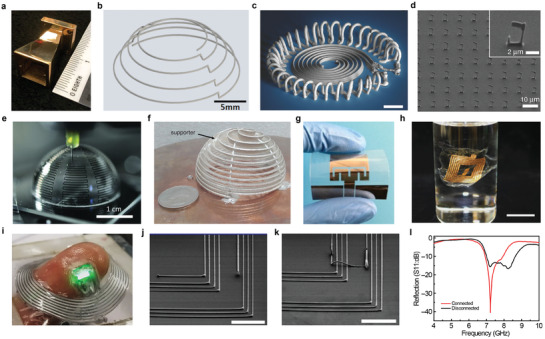
3D‐printed antennas. a) Photograph of 3D‐printed copper horn antenna. b) Schematic illustration of the 3D shaped spiral antenna. c) Printed LC resonator consisting of a 32‐turn toroidal inductor and a parallel‐plate capacitor. Scale bar, 100 µm. d) SEM image of an array of vertical SRRs, with a period of 9.2 µm. Inset: Close‐up of a single vertical SRR. e) Optical image of an antenna during the conformal printing process. f) Photograph of fabricated strip‐FSH antenna with electrically small size. g) Photograph of the printed coplanar waveguide feed antenna on PET substrate. h) Photograph of graphene/gold bilayer spiral antenna coil on polymer nanosheet. Scale bar, 1 cm. i) Photograph of wireless light‐emitting device based on liquid metal antenna. Embedded LED chips were wirelessly turned on when the antenna was deformed by a finger. j) SEM image of the printed inner/outer coil antenna. Scale bar, 300 µm. k) SEM image of connected lines by reconfiguration. Scale bar, 300 µm. l) Measured scattering parameters of the printed antenna in disconnected and connected states. a) Reproduced with permission.^[^ ^167^ ^]^ Copyright 2015, MDPI. b) Reproduced with permission.^[^ ^170^ ^]^ Copyright 2018, Wiley‐VCH. c) Reproduced with permission.^[^ ^171^ ^]^ Copyright 2017, Wiley‐VCH. d) Reproduced with permission.^[^ ^95^ ^]^ Copyright 2021, Springer Nature. e) Reproduced with permission.^[^ ^173^ ^]^ Copyright 2011, Wiley‐VCH. f) Reproduced with permission.^[^ ^174^ ^]^ Copyright 2016, The Institution of Engineering and Technology. g) Reproduced with permission.^[^ ^175^ ^]^ Copyright 2017, Wiley Periodicals, Inc. h) Reproduced with permission.^[^ ^176^ ^]^ Copyright 2019, Wiley‐VCH. i) Reproduced with permission.^[^ ^178^ ^]^ Copyright 2021, Wiley‐VCH. j‐l) Reproduced with permission.^[^ ^9^ ^]^ Copyright 2019, American Association for the Advancement of Science.

Compared to the conventional antenna manufacturing method, the advantages of antenna 3D printing technology include the extensive range of ink materials, complex structural design, minimized waste of material, and fast turnaround time. Due to these advantages, many studies have been conducted to fabricate antennas through 3D printing. Zhang et al. fabricated copper and aluminum alloy horn antennas through metallic 3D printing.^[^ ^167^ ^]^ Two horn antennas were printed by selective laser melting (SLM) technology of copper (85% copper and 15% tin) and aluminum alloy (89.5% aluminum, 10% silicon, and 0.5% magnesium), respectively. The printed metal antenna both have an impedance bandwidth across the K‐band. In addition, the gains of copper and aluminum alloy horn antennas are 13.23 dBi at 25 GHz and 13.5 dBi at 24 GHz, respectively, proving that they can replace the conventional antenna (**Figure** **10**a). Tak et al. fabricated a lightweight X‐band waveguide horn antenna through 3D printing and conductive spray coating.^[^ ^168^ ^]^ The horn antenna was produced through fused deposition modeling (FDM)‐based 3D printing of polylactic acid (PLA) and metalized through commercial conductive spray (Kontakt Chemie, EMI 35). The 3D‐printed horn antenna weighed 19.3% of the weight of the metallic antenna made by the conventional method. It also proved that the values of the gain and reflection coefficient are similar to those of the conventional antenna. McKerricher et al. manufactured a fully printed antenna by inkjet printing plastic material and silver conductor.^[^ ^169^ ^]^ The substrate of the antenna was made of UV‐curable polymer, and the antenna was selectively laser sintered with silver nanoparticles. The obtained 2.4 GHz patch antenna showed a gain of 8 dBi and radiation efficiency of 81%. Considering that the gain and radiation efficiency of the solid substrate antenna were 5.8 dBi and 67%, respectively, it was demonstrated that the inkjet process improved the characteristics of the antenna. Kim et al. manufactured a 3D spiral antenna by direct writing of nanocellulose‐based ink.^[^ ^170^ ^]^ Printable ink was fabricated by mixing silver nanowires as a conductivity filler, and cellulose nanofibers (CNFs) as a matrix material. Optimized ink was 3D‐printed on the hemisphere surface to form a spiral antenna, by direct writing. The resonance occurs at 2.48 GHz where the reflection coefficient shows the minimum and the real part of impedance shows the maximum (Figure 10b). Zhou et al. demonstrated in‐plane and out‐of‐plane passive RF freeform structures by direct writing of silver nanoparticles.^[^ ^171^ ^]^ An inductor‐capacitor (LC) resonator consisting of a 32‐turn toroidal inductor and parallel‐plate capacitor was printed with a line width of 10 µm or below (Figure 10c). The LC resonator exhibits resonance frequency of 6.5 GHz where magnitude of input impedance was a maximum and quality factor (Q) of 10. Jung et al. developed 3D nanoprinting technology using charged aerosol and fabricated 3D freeform plasmonic structures such as vertical split‐ring resonators (SRRs) of gold.^[^ ^95^ ^]^ Unlike the conventional printing method that uses polymer or ink, this method used charged aerosols as a building block. The fabricated vertical SRR showed high resolution with line width below 2 µm. The magnetic resonance of the vertical SRR revealed a dip at resonance wavelength of *λ*_res_ = 19.5 µm in both simulation and measurement results. The surface current of the vertical SRR formed a magnetic dipole moment that was parallel to the substrate, demonstrating the possibility that it can be applied as an antenna (Figure 10d).

Due to the design limitations caused by the recent miniaturization of wireless devices, further miniaturization of the antenna is required. The electrical size of the antenna is defined as *ka*, where *k* is the wavenumber and *a* is the radius of the sphere surrounding the maximum size of the antenna. In general, according to the theory of Wheeler and Chu, electrically small antennas (ESAs) have an electrical size defined as *ka* ≤ 0.5.^[^ ^172^ ^]^ Since the ESA has a high reactance value and a very small radiation resistance value, impedance matching is difficult. Therefore, the ESA has a very narrow impedance bandwidth, low efficiency, and has a low gain. To overcome these limitations, studies are underway to design ESA with high performance and small size using 3D printing. Adams et al. demonstrated an ESA through conformal printing of silver nanoparticle inks.^[^ ^173^ ^]^ Proposed ESAs were fabricated by conformal printing of silver nanoparticle ink in the form of meander lines on convex and concave hemispherical surfaces. The printed meander lines showed a line width of about 100 µm. The center frequency of the fabricated ESAs was at 1.73 GHz with bandwidth and efficiency of 15.2% and 71%, respectively which demonstrates that the performance approaches the Chu limit (Figure 10e). In another form of ESAs, Kong et al. proposed volumetric‐folded spherical helix (FSH) antennas.^[^ ^174^ ^]^ The structure of the antenna was fabricated through selective laser sintering (SLS) printing of polyamide material and painted with silver paste to have sufficiently high conductivity. The ka value of the printed volumetric FSH antenna is 0.21, which is an electrically small antenna, and the resonance occurs at 307 MHz with the radiation efficiency of 90%, demonstrating the good agreement with the simulation results (Figure 10f).

For application to flexible electronics and wearable devices, studies on antennas that maintain characteristics even in bending or stretching environments are in progress. Guo et al. proposed a fully printed, flexible, reversibly deformable coplanar waveguide feed (CPW) antenna.^[^ ^175^ ^]^ Silver nanoparticles were printed on a flexible PET substrate by an inkjet printer to constitute the radiating element and the ground plane. The simulated and measured reflection coefficients of the fabricated antenna are ≈31 and ≈23 dB, respectively. Reflection coefficient in bending environment revealed the negligible shift of center frequency (Figure 10g). Tetsu et al. developed an ultra‐flexible antenna coil through graphene/Au hybrid inkjet printing.^[^ ^176^ ^]^ The graphene/gold bilayer spiral antenna coil is 24.96 cm in length and 5.5 turns in total, which was inkjet‐printed into the glass substrate and transferred to polymer nanosheets, which is possible because graphene flakes are piled up with weak Van der Waals forces.^[^ ^177^ ^]^ The electro‐mechanical properties of the nanosheet antenna coil were evaluated through bending tests. The resistance of the antenna coil was constant at radius bending of 5, 9, and 14 mm, and was also constant at 100 bending cycles test. The frequency characteristic of the antenna coil also showed that the resonance frequency was stable at 20 MHz in different curvatures of 5–14 mm (Figure 10h). Yamagishi et al. fabricated a thin film‐based liquid metal injected microfluidic channel antenna with high deformability.^[^ ^178^ ^]^ The authors formed a coil‐shaped silicone sealant microfluidic channel with a channel width of 67 µm, through direct writing on flexible/stretchable Ecoflex substrates which were followed by the injection of gallium‐based liquid metal in the microfluidic channel. The proposed wireless light‐emitting device is operated by a standard near‐field‐communication (NFC) system (13.56 MHz) and maintained the same performance under deformations including stretching (>200% uniaxial strain), twisting (180°) and bending (3.0 mm radius of curvature). And it also sustained a high quality factor (*Q* > 20) verifying high wireless powering efficiency (Figure 10i). Park et al. proposed a reconfigurable liquid metal antenna capable of modifying the resonance frequency and radiation properties.^[^ ^9^ ^]^ The inner/outer coil antenna was initially formed by direct printing of eutectic gallium–indium alloy (EGaIn) (Figure 10j). The reconfiguration process of printed EGaIn formed a 3D bridged interconnects connecting the inner coil and the outer coil, resulting in a single coil with an increased antenna turn number (Figure 10k). The frequency response of the reflection coefficient of the dual coil showed a double peak, whereas the 3D bridged single coil antenna showed a narrow bandwidth with a higher reflection efficiency due to an increase in the total number of turns (Figure 10l).

### 4.4 Sensors

**Figure 11:**
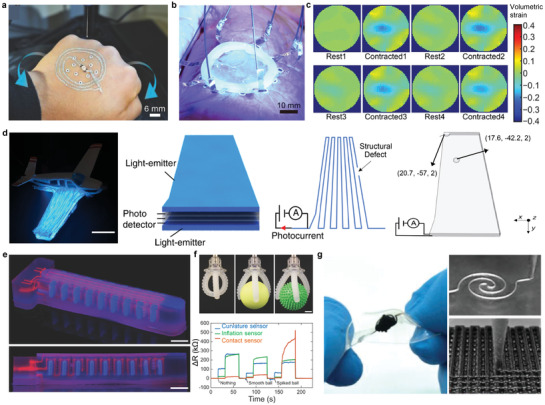
3D‐printed sensors. a) Photograph of adaptive 3D printing of the wireless moisture sensor on a human hand that can move freely in the workspace. b) Photograph of deformation sensors printed on a porcine lung. c) Spatiotemporal mapping of volumetric strain within the region‐of‐interest on a porcine lung undergoing cyclic contraction. d) Application of a printed bi‐functional light‐detecting and light‐emitting 3D‐structure for detecting structural defects of model airplane. Scale bar, 2 cm. e) Fluorescence image of integrated sensory robotic gripper. The actuator (blue) and sensor (red) inks have been fluorescently dyed to facilitate visualization. Scale bars, 10 mm. f) Top: Photographs showing the gripper holding nothing (left), a smooth ball (middle), and a spiked ball (right). Scale bar, 20 mm. Bottom: Resistance change of curvature, inflation, and contact sensors as a function of time. g) Photographs of 3D‐printed piezoresistive sensor and its magnified views of electrode layer and sensing layer. a) Reproduced with permission.^[^ ^58^ ^]^ Copyright 2018, Wiley‐VCH. b,c) Reproduced with permission.^[^ ^187^ ^]^ Copyright 2020, American Association for the Advancement of Science. d) Reproduced with permission.^[^ ^188^ ^]^ Copyright 2019, Springer Nature. e,f) Reproduced with permission.^[^ ^189^ ^]^ Copyright 2018, Wiley‐VCH. g) Reproduced with permission.^[^ ^190^ ^]^ Copyright 2019, Wiley‐VCH.

As the measurement of environmental parameters is one of the most important necessities in our essential life, various types of sensors have been developed. Sensors for measuring strain, pressure, temperature, chemicals, and biomarkers can be applied to vast fields including industrial maintenance, wearable healthcare, and IoT.^[^ ^179^, ^180^, ^181^, ^182^ ^]^ According to the progress of sensor technologies and the increase in applicability of sensors, the structure of sensors is required to conform to 3D arbitrary environments or objects. Especially, intensive research in wearable biosensors for medical application concentrates to design the structures to fit along the biological surfaces.^[^ ^183^, ^184^ ^]^ Therefore, 3D printing technologies have been actively used to form sensors with 3D and conformable structures. Zhu et al. demonstrated the fabrication of tactile sensors on arbitrary surfaces using 3D direct writing of silver nanocomposite inks.^[^ ^185^ ^]^ the extrusion‐based direct writing was done under ambient conditions. The tactile sensor was directly printed on the human skin and detected the movements including pulses and finger motions. For further study, Zhu et al. presented the 3D‐printed moisture sensors on human skin.^[^ ^186^ ^]^ This extrusion‐based 3D direct writing equipped the closed‐loop tracking system, of which nozzles could track the motion of target surfaces. The fiducial markers were placed around the hand surface, and the nozzle tracked the markers and printed moisture sensors during the movement of the hand (**Figure** **11**a). Using this adaptive 3D printing, deformation sensors were conformally printed on the surface of the porcine lung (Figure 11b).^[^ ^187^ ^]^ As a material for deformation sensors, the ionic hydrogel was used for strain‐sensitive printable material. After printing of hydrogel within region‐of‐interest on a porcine lung, the spatial mapping of deformation was measured by electrical impedance tomography using multiple copper electrodes placed around the hydrogel (Figure 11c).

3D‐printed sensors can be applied to the industrial field for monitoring the defect of 3D objects or machines with conformal contact. Loke et al. presented the integrated light‐emitting and light‐detecting 3D structures through direct writing‐based multi‐nozzle printers.^[^ ^188^ ^]^ Light‐emitting and light‐detecting parts were a form of filament made by multiple inks of metals, dielectrics, and semiconductors. Each filament was printed through separate nozzles simultaneously and formed the integrated 3D structures (Figure 11d). Using this method, this 3D structure was applied to detect structural defects of a wing of a model airplane.

The increased degree of freedom in fabricating the sensor takes advantage of their integration to heterogeneous devices. Truby et al. demonstrated all‐printed robotic grippers with the integration of haptic and temperature sensing compartments.^[^ ^189^ ^]^ The printing was done by the commercial direct writing‐based 3D printer. The ink for actuators (blue fluorescence in Figure 11e) was made of platinum‐cure silicone elastomer, and the ink for sensors (red fluorescence in Figure 11e) was made of organic ionic liquid 1‐ethyl‐3‐methylimidazolium ethyl sulfate (EMIM‐ES) filled with fumed silica particles as rheological modifiers. By grabbing objects with different textures, resistance changes in ionic liquid‐based sensors were measured (Figure 11f). Wang et al. presented a piezoresistive sensor with soft materials.^[^ ^190^ ^]^ As an ink for soft, stretchable piezoresistive material, carbon black‐polyurethane elastomer composite with NaCl inclusion (Figure 11g). After 3D direct writing, NaCl in the composite was removed by water, which modified the sensitivity by generating porosity in printed 3D structures.

## 5 Perspectives

As the miniaturization of electronic devices becomes progressed further, they are required to provide more complex shapes with offering multiplexed functions. Especially, the formation of freeform 3D electronic devices using multiple functional materials is essential for providing conventional electronic systems an extra degree of design with high device integrity. However, the conventional photolithography‐based microfabrication techniques require multiple fabrication steps, including vacuum depositions or etchings that have high fabrication costs and cause unnecessary waste of materials. In addition, the photolithography is available only for 2D structures on a planar surface. Therefore 3D printing methods can be a good breakthrough to overcome the limitations of photolithography. Although commercially available 3D printing techniques are useful for many applications, their limits in printable materials, printing resolutions, or processing conditions are significant challenges in achieving freeform 3D electronics, as explained in the below. In this section, we describe the limitations of current 3D printing methods, with suggesting ways to improve and visions.

### 5.1 Scalability

#### 5.1.1 Slow Printing Speed

Currently, many 3D printers are lagging in speed and efficiency compared to traditional production equipment. Slow printing speed is an obstacle to their application in industries that require mass production, so improving the speed of 3D printing is required to maintain production efficiency at a similar level to the conventional process. In particular, if the printing speed is accelerated further, 3D printing methods can be used to fabricate fully integrated electronics systems including the sensors and interconnects with complex architectures.

To solve this problem, Liashenko et al. presented a strategy to enable a fast printing process in nozzle‐based 3D printing techniques, based on the control of the trajectory of an electrified jet through rapidly tuning the surrounding electrostatic field through additional electrodes. The printing speed achieved up to 0.5 m s^−1^.^[^ ^191^ ^]^

#### 5.1.2 Mismatch of Material Properties

3D printing technology is demanded as a solution for diverse materials. 3D printing can theoretically produce highly complex and functional parts regardless of the mechanical, biological, or electrical properties of the material. However, to make it possible, it is necessary to select appropriate material and process it to enable printing. Compared to traditional manufacturing processes that have undergone decades of development in processible materials, the research for 3D‐printable materials is a beginning step. Although numerous studies have been conducted, there are still many mismatches between the properties of 3D printable materials and printing technology. For various applications in wearable electronics, bioelectronics, or soft robotics, the development of 3D‐printable functional materials and the control of ink properties are essentially needed. For example, 3D printing of a battery can raise safety issues, if the properties of printable ink materials do not match. Currently, there is a lack of a validated database of parameters for printing and properties of materials.

As a result, it becomes difficult to achieve a consistent and repeatable 3D printing process. To overcome this problem, recent studies have been conducted to predict the properties of printing materials through various simulation programs.^[^ ^192^ ^]^

#### 5.1.3 Pre‐ and Post‐processing

Almost all 3D‐printed objects require post‐processing to improve their mechanical properties, accuracy, and esthetics. When the printed part is removed from the plate, the accumulated residual heat can distort the part during cooling. It may be more efficient to use human labor for dozens of levels of prototypes, but when producing thousands of 3D‐printed parts, it is necessary to automate the recovery, cleaning, grinding, and post‐processing to remove supports and powders. As part of a method to reduce the time and cost of pre‐processing work, research on software that facilitates 3D modeling is being progressed.^[^ ^193^ ^]^

### 5.2 Miniaturization

For the implementation of high‐performance electronics, complexity and device density in a 3D form have increased. Therefore, miniaturization of 3D‐printable electronic structures is required.^[^ ^194^ ^]^ Existing micro‐scale electronics, including the integrated internal circuits and components, are being miniaturized to the submicron to nanoscale. For the application of 3D printing to such miniaturized electronics (e.g., interconnect, antenna, battery), high‐resolution printing has become one of the key challenges for developing electronics. However, to date, 3D printing technology has technical limitations in aligning nanosized nozzles that determine the diameter of printed structures or 3D printing of ink materials at the submicron scale.

Studies are being conducted actively to overcome these limitations and perform high‐resolution printing. Bae et al. embedded a quantum dot corresponding to RGB in polystyrene nanowire to produce a nanophotonic ink.^[^ ^195^ ^]^ Direct writing by a glass nanopipette nozzle with an opening diameter of 630 ± 70 nm was performed to print a 3D nanopillar structure. Without the change in the spatial resolution of the pixel, an increase in the brightness of the pixel was made by the increase of the pillar height indicating the availability to manufacture a high‐resolution display device without changing the spatial resolution of the pixel. While there is a method by making the nanoscale nozzle, studies for printing miniaturized 3D structures utilizing other components such as electric fields have been developed.^[^ ^196^ ^]^ Park et al. formed a nanowall with a high aspect ratio using a 3D near‐field electrospinning technique.^[^ ^197^ ^]^ Unlike the existing uncontrollable e‐jet printing method, a controllable e‐jet printing system was built by reducing the distance and applied voltages between the nozzle and the collector. Adding NaCl salt to poly(ethylene oxide) solution further increased the interaction between the nanofibers and allowed the precise control to the z‐axis and fiber deposition layer‐by‐layer from micro to nanoscale. Jung et al. developed an nm scale palladium (Pd) nanopillar array by using a charged aerosol jet printing method.^[^ ^95^ ^]^ By positioning a micro‐sized hole (4 mm) SiN*_x_* mask utilized as a nozzle, electrostatic focusing of the charged aerosol could be performed to form a 3D metal with a submicron scale (≈300 nm). An et al. demonstrated the high‐resolution 3D printing of complex 3D structures by electrohydrodynamic inkjet printing.^[^ ^94^ ^]^ They have fabricated complex geometry such as vertical or helix‐shaped pillars, rounded walls, and bridge‐like structures using silver, copper, and cobalt.

The resolution of 3D printing technologies has reached to the scale of submicrons. Compared to the current state‐of‐the‐art electronics based on the nanometer‐scale photolithography, the 3D printing resolution should be enhanced further. For nozzle‐based technologies, such as ink‐jet and direct writing, the formation of fine nozzle arrays and the nanoscale manipulation of printing motions must be accompanied together. For high‐resolution and selective photocuring, a high‐intensity focus of photons in nanoscales is also required. Furthermore, in order to prevent the unexpected curing that can lower printing resolutions, the light intensity control to minimize the interval between focusing and unfocusing needs to be optimized as well.

### 5.3 High‐Aspect‐Ratio Structures

The continued decrease in the size of electronics requires a higher resolution of 3D printing technology. Especially, in the case of a conductive pathway that connects circuits, a high‐aspect‐ratio structure is required as the line width decreases. Also, as the complexity of the components within the limited area increases, high aspect ratios of devices for efficient designs become strongly demanded.^[^ ^198^ ^]^ However, to date, 3D printing technology has had a limit in meeting the need for such a high‐aspect‐ratio 3D structure. Therefore, studies are underway to overcome these limitations.

Chu et al. proposed a metal line fabrication method with a high aspect ratio through the control of ink drying dynamics and interaction between silver ink and the substrate.^[^ ^199^ ^]^ The authors formed silver nanoparticle‐based ink on a SU‐8 coated PET substrate through inkjet printing which was followed by heat treatment to induce contraction. Through this contraction process, a silver line was formed with a high aspect ratio. This method reduced the width of inkjet‐printed Ag lines more than ten times and increased the thickness‐to‐width aspect ratio. Sowade et al. formed metal pillars of the high aspect ratio through inkjet printing, which can be used as vertical interconnects.^[^ ^145^ ^]^ Silver nanoparticle ink was deposited layer by layer through inkjet printing to form a pillar shape. The height of the pillar increased in proportion to the number of printed layers, and the maximum height of a pillar was about 10 mm, and the aspect ratio was 50:1. Schneider et al. proposed complex metal‐grid structures with high aspect ratios using electrohydrodynamic printing.^[^ ^200^ ^]^ They tuned the droplet ejection and vaporization rate of inks to stack up the printed layers vertically. In this way, metallic walls were printed using silver or gold colloidal inks with the line‐width from 80 to 500 nm and the height from 200 nm to 1.5 µm. Here the maximum aspect ratio was >7.

Even materials with similar properties can offer different aspect ratios through adjustments of printing dynamics and drop drying mechanisms. Therefore, it is necessary to accumulate data by conducting prior research on how the aspect ratio can vary for each material under different conditions. Based on these studies, the groundwork for realizing high aspect ratios can be prepared.

### 5.4 Multifunctional Materials

To realize electronic device system by 3D printing, a variety of materials should be integrated at the same time. However, only a single or a few materials can be printed at once with most of the 3D printing technologies widely used nowadays. In 3D printing technologies, the printability of multiple materials with a single printing process is required to make it possible for fabricating integrated electronics.^[^ ^201^ ^]^

Skylar‐Scott et al. proposed a direct writing method that enables rapid switching between different materials printed through a single nozzle.^[^ ^202^ ^]^ The authors were able to fabricate 2D arrays of printheads that can print up to eight different materials which were operated at a switching frequency of up to 50 Hz, enabling the formation of multi‐material 3D structures. A soft‐robotic walker's legs were printed from the aforementioned printing method and with two different silicone rubbers of different stiffnesses. Using multiple parallel nozzles reduced the time of printing, which is important for both the scalability of fabrication and the stability of some printable materials which started to harden once it was printed. Chen et al. introduced a way of electrohydrodynamic printing method that offers simplicity in multi‐material, parallel 3D printing.^[^ ^203^ ^]^ A double‐barreled glass nanopipette and silicon substrate were used for the printing process. By using this printing method and Ag ink and CdSe/ZnS quantum dot (QD) ink as the printing materials, the parallel fabrication of nanomeshes and nanowalls that was composed of a mixture of Ag and QDs were demonstrated.

Simultaneous in‐situ printing of multiple functional materials is a key challenge in current 3D printing methods, as in the examples presented. Thus, developing a variety of functional materials for high‐performance electronic devices is essential, and next‐generation 3D printing technologies should proceed with the diversification of printable materials to enlarge their application areas.

## 6 Conclusion

Unprecedented revolutions in material designs and printing methods to form 3D electronic structures have led to enormous progress in the fabrication technologies for various electronic devices. The diversity of printable electronic materials from insulators and semiconductors to conductors, hard inorganics to soft organics, enables the heterogeneous integration of various device components with disparate mechanical, electrical, and optical properties in arbitrary spaces. Also, numerous 3D printing methods provide new architectures of materials under a mild and ambient condition with additive manufacturing, minimizing the damage to substrates and surrounding environments.

The complexity and integration of current 3D‐printed electronics are relatively simplistic, and in some cases, they run behind conventional photolithography‐based 2D electronics. Their improvement can be achieved by developing multi‐material printing systems with minimized positioning and switching errors. In addition, to be the future 3D electronics with high performances comparable to conventional 2D electronics, the structural scales should be controllable from the nanoscale to the microscale. For industrial applicability, scalable fabrication using 3D printing should be achieved, and the rapid production of high‐resolution patterns can be realized by high‐throughput systems such as multi‐nozzle 3D printing.

Meanwhile, the rapid growth of freeform electronic devices starting with wearable electronics will require more intimate, conformal forms to various body parts of individual users. To accord with the needs, 3D printing technologies should respond to the versatility in material usage from electronic functional materials to soft biomaterials, to be applied for biomedical and healthcare purposes. Also, the biocompatibility of electronic ink material and low‐temperature processes are key points to be considered. Many of the seemingly critical challenges in 3D printing technologies addressed a few years ago are orderly being solved by steady and intensive research of the field. We believe these efforts will lead us into the era of 3D‐printed electronics presently.

## Conflict of Interest

The authors declare no conflict of interest.
